# The Forkhead box F1 transcription factor inhibits collagen deposition and accumulation of myofibroblasts during liver fibrosis

**DOI:** 10.1242/bio.039800

**Published:** 2019-01-23

**Authors:** Hannah M. Flood, Craig Bolte, Nupur Dasgupta, Akanksha Sharma, Yufang Zhang, Chandrashekhar R. Gandhi, Tanya V. Kalin, Vladimir V. Kalinichenko

**Affiliations:** 1 Department of Pediatrics, Cincinnati Children's Research Foundation, Cincinnati, Ohio 45229, USA; 2 Division of Human Genetics, Cincinnati Children's Research Foundation, Cincinnati, Ohio 45229, USA; 3 Division of Gastroenterology, Hepatology, and Nutrition, Cincinnati Children's Research Foundation, Cincinnati, Ohio 45229, USA

**Keywords:** FOXF1, Hepatic fibrosis, Myofibroblast, Hepatic stellate cell, Carbon tetrachloride liver injury

## Abstract

Hepatic fibrosis is the common end stage to a variety of chronic liver injuries and is characterized by an excessive deposition of extracellular matrix (ECM), which disrupts the liver architecture and impairs liver function. The fibrous lesions are produced by myofibroblasts, which differentiate from hepatic stellate cells (HSC). The myofibroblast’s transcriptional networks remain poorly characterized. Previous studies have shown that the Forkhead box F1 (FOXF1) transcription factor is expressed in HSCs and stimulates their activation during acute liver injury; however, the role of FOXF1 in the progression of hepatic fibrosis is unknown. In the present study, we generated *αSMACreER;Foxf1^fl/fl^* mice to conditionally inactivate *Foxf1* in myofibroblasts during carbon tetrachloride-mediated liver fibrosis. *Foxf1* deletion increased collagen depositions and disrupted liver architecture. *Timp2* expression was significantly increased in *Foxf1*-deficient mice while MMP9 activity was reduced. RNA sequencing of purified liver myofibroblasts demonstrated that FOXF1 inhibits expression of pro-fibrotic genes, *Col1α2*, *Col5α2*, and *Mmp2* in fibrotic livers and binds to active repressors located in promotors and introns of these genes. Overexpression of FOXF1 inhibits *Col1a2*, *Col5a2*, and *MMP2* in primary murine HSCs *in vitro*. Altogether, FOXF1 prevents aberrant ECM depositions during hepatic fibrosis by repressing pro-fibrotic gene transcription in myofibroblasts and HSCs.

## Abstract (precis)

**Summary:** The transcription factor FOXF1 is expressed in liver fibroblasts and regulates fibrosis by repressing pro-fibrotic genes. FOXF1 prevents aberrant extracellular matrix depositions during hepatic fibrosis.

## INTRODUCTION

The liver is the body's filter and insults can result from a variety of infectious, toxic and metabolic agents. Hepatic fibrosis is the common end stage to a multitude of liver diseases (Civan, 2016) and is characterized by an excessive deposition of extracellular matrix (ECM) and collagen (Cheng and Mahato, 2007). Novel animal models of hepatic fibrosis are greatly needed to identify molecular mechanisms responsible for the disease pathogenesis and for the development of therapeutic agents. Hepatic stellate cells (HSC) reside in the space of Disse and are characterized by their storage of lipids when in the quiescent state (Yin et al., 2013; Croci et al., 2013). During fibrogenesis, quiescent HSCs differentiate into myofibroblasts (MF) in response to cytokine signaling from damaged hepatocytes and immune cells after liver insult. MFs secrete ECM and collagen to encapsulate the site of injury and shield the liver from plaguing insults (Cheng and Mahato, 2007). While HSCs and MFs make up only a small number of cells in liver tissue, they are the main contributors of ECM and collagen during liver repair and fibrogenesis (Brenner et al., 2012; Fausther et al., 2013). The TGF-β and PDGF signaling pathways play key roles in hepatic fibrosis and HSC activation (Makarev et al., 2016). TGF-β signaling stimulates cellular transdifferentiation of HSCs to MFs (Hellerbrand et al., 1999; Bachem et al., 1993), whereas PDGF signaling induces cellular proliferation in fibrotic foci (Wong et al., 1994; Kinnman et al., 2002).

The Forkhead Box F1 (FOXF1) transcription factor is expressed in human and murine HSCs and is important in regulating stellate cell activation after acute liver injury (Kalinichenko et al., 2003). In the advanced disease state of hepatocellular carcinoma (HCC), which is associated with significant fibrotic depositions, *FOXF1* expression has been shown to be significantly decreased (Hodo et al., 2013). *Foxf1^−/−^* mice are embryonic lethal due to severe developmental abnormalities in the yolk sac and allantois (Mahlapuu et al., 2001). Murine haploinsufficiency of *Foxf1* causes lung hypoplasia, loss of alveolar capillaries in the lung and gall bladder agenesis (Kalinichenko et al., 2002; Bolte et al., 2018), and was associated with delayed lung and liver repair. After acute liver injury by carbon tetrachloride (CCl_4_), *Foxf1*^+/−^ mice exhibited diminished activation of HSCs and delayed liver repair, indicating that FOXF1 is essential for liver repair after acute liver injury (Kalinichenko et al., 2003). *Foxf1* siRNA delivered to mice through nanoparticles prevented activation of HSCs and subsequent collagen deposition after cholestatic liver injury (Abshagen et al., 2015). While these studies have shown that FOXF1 is required for activation of HSCs after acute liver injury, the role of FOXF1 in MFs and in the progression of fibrotic responses remains unknown.

In the present study, we generated a novel genetic mouse model to conditionally delete *Foxf1* from MFs (*αSMACreER;Foxf1^fl/fl^*). During chronic liver injury, deletion of *Foxf1* in MFs exacerbated hepatic fibrosis, increased collagen deposition and stimulated expression of profibrotic genes in the liver tissue. Our studies indicate that *Foxf1* expression in MFs is critical to prevent MF accumulation and collagen deposition during liver fibrosis.

## RESULTS

### Deletion of *Foxf1* in αSMA-positive cells exacerbates CCl_4_-induced hepatic fibrosis

**Fig. 1.**
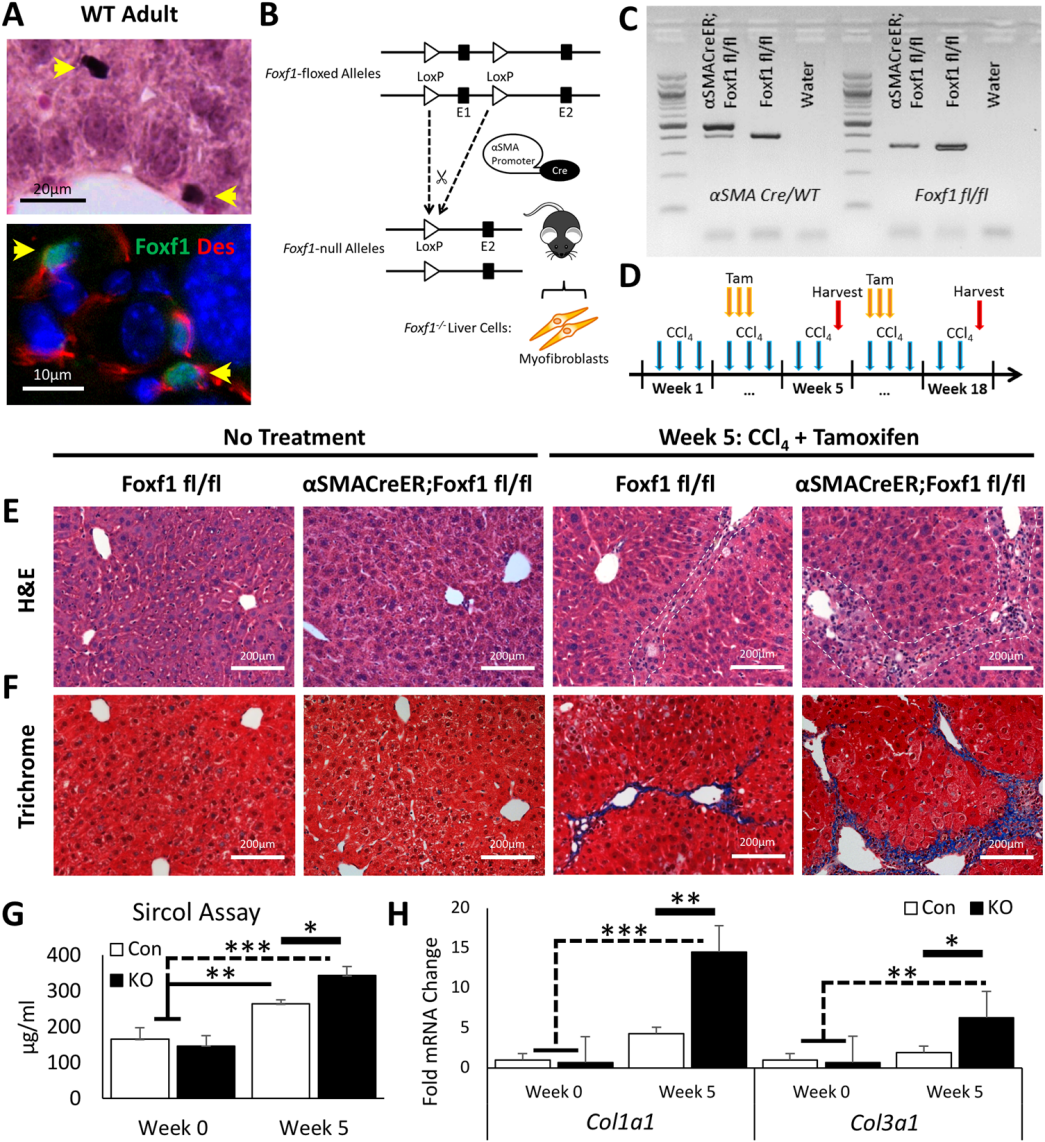
**Hepatic fibrosis is increased after CCl_4_ injury in mice with FOXF1 deficiency.** (A) FOXF1 co-localizes with DES in hepatic stellate cells in adult mice. (B) Diagram demonstrates *αSMA-CreER* transgene with LoxP sites flanking the *Foxf1* Exon 1 (encoding DNA-binding domain). (C) DNA gel shows genotypes of *Foxf1^fl/fl^* and *αSMACreER;Foxf1^fl/fl^* mice. (D) Diagram illustrates CCl_4_ and tamoxifen (Tam) treatment protocol. (E,F) H&E and Masson's trichrome staining show fibrotic depositions after five weeks of CCl_4_ treatment. Fibrosis was increased in livers from *αSMACreER;Foxf1^−/−^* mice. White dashed lines indicate fibrotic lesion boundaries. (G) Collagen deposition was quantitated using the Sircol assay. *n*=2 mice per group in week 0; *n*=4 mice per group in week 5. (H) qRT-PCR analysis demonstrates significant increases in *Col1α1* and *Col3α1* mRNAs in livers from *αSMACreER;Foxf1^−/−^* mice. *n*=3 mice per group in week 0; *n*=5 mice per group in week 5. Untreated livers from *Foxf1^fl/fl^* and *αSMACreER;Foxf1^fl/fl^* mice were used as normal controls. mRNAs were normalized to *Actb*. *P<0.05, **P<0.01, ***P<0.001.

Previous studies demonstrated that FOXF1 is present in HSCs in murine developing and adult livers (Kalinichenko et al., 2003; Kim et al., 2005). Consistent with these studies, FOXF1 staining was detected in livers of e12.5-e17.5 mouse embryos as well as in mesenchyme of stomach and intestine (Fig. S1). In adult mice, FOXF1 is specifically expressed in the liver parenchyma but not in endothelial or smooth muscle cells surrounding the portal vein or hepatic artery (Kalinichenko et al., 2003) (Fig. 1A; Fig. S1), and FOXF1 staining co-localized with desmin (DES) (Fig. 1A), a known marker of HSCs (Yokoi et al., 1984). To investigate the role of *Foxf1* in liver fibrosis, we utilized a conditional knockout approach. Transgenic mice containing a tamoxifen-inducible *αSMA-CreER* transgene and two *Foxf1*-floxed alleles (*αSMACreER;Foxf1^fl/fl^*) were generated by breeding *αSMA-CreER* and *Foxf1^fl/fl^* mice (Fig. 1B,C). Hepatic fibrosis was induced by chronic liver injury using multiple administrations of CCl_4_, which is known to increase fibrotic depositions and disrupt liver architecture in experimental mice (Martinez et al., 2014). Tamoxifen was given three times per week to achieve a continuous deletion of *Foxf1* in αSMA-positive MFs (Fig. 1D) that derive from HSCs after liver injury (Mederacke et al., 2013). Morphological analysis of liver sections revealed increased fibrotic deposition in CCl_4_-treated *αSMACreER;Foxf1^−/−^* livers compared to controls as shown by H&E (Fig. 1E; Fig. S2) and Masson's Trichrome staining (Fig. 1F; Fig. S2). Increased fibrosis in *αSMACreER;Foxf1^−/−^* livers was confirmed by significant increases in collagen levels by Sircol (Fig. 1G) and hydroxyproline (Fig. S2) assays as well as by qRT-PCR for *Col1α1* and *Col3α1* mRNAs (Fig. 1H). Treatment with tamoxifen alone (without CCl_4_) did not affect liver architecture or induce liver fibrosis (Fig. S2). Thus, deletion of *Foxf1* from MFs accelerates liver fibrosis after chronic liver injury.

### FOXF1 expression is decreased in hepatic myofibroblasts of *αSMACreER;Foxf1^−/−^* mice

**Fig. 2.**
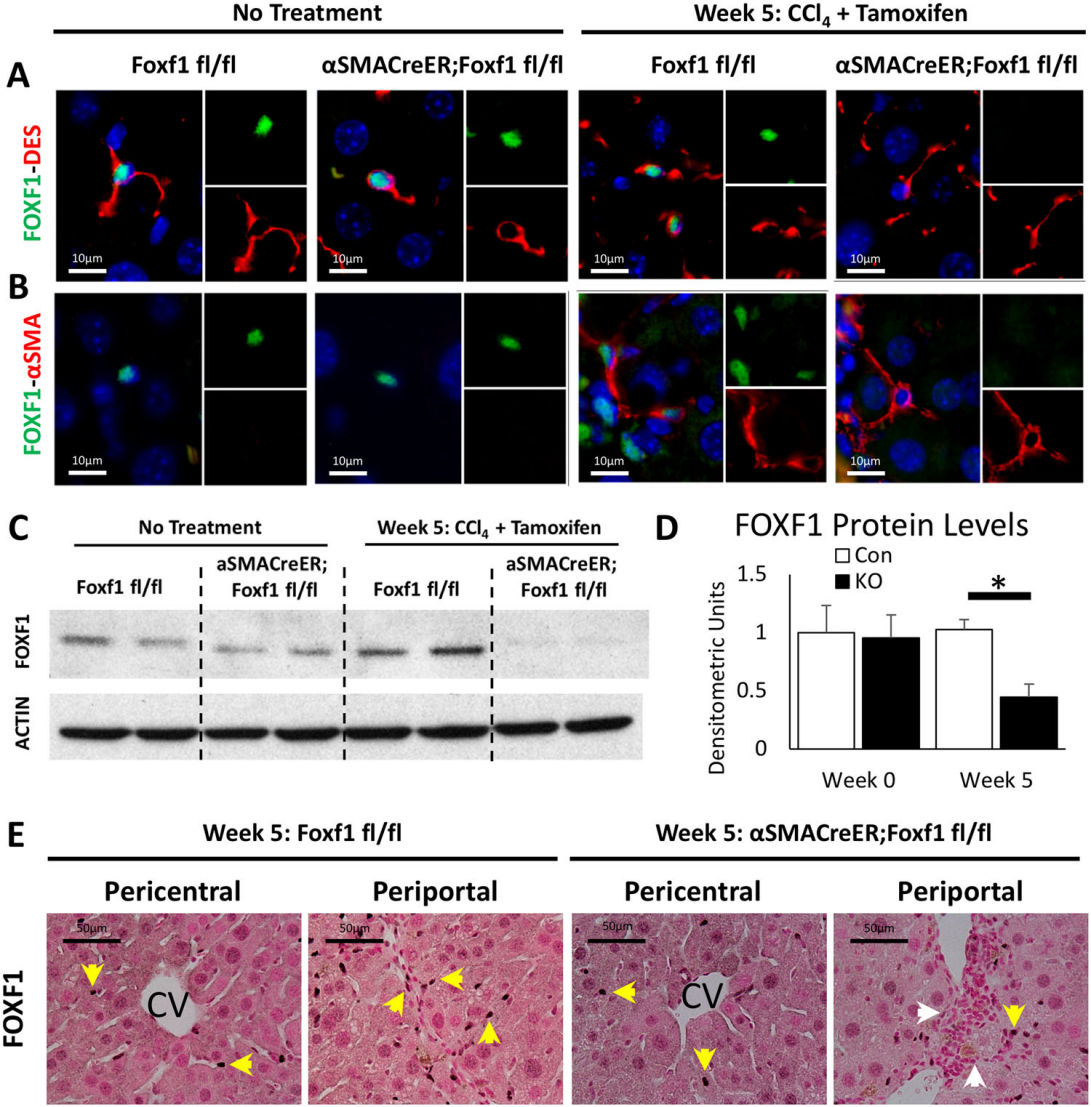
**αSMA-CreER effectively deletes *Foxf1* from hepatic myofibroblasts.** (A,B) FOXF1 co-localizes with DES in HSCs before and after CCl_4_-induced injury. FOXF1 co-localizes with DES and αSMA in MFs after chronic liver injury. *αSMA-CreER* effectively deletes *Foxf1* from MFs after Tam treatment. (C) Western blot shows total liver protein levels of FOXF1 are decreased in *αSMACreER;Foxf1^−/−^* livers after CCl_4_ injury. Cropped blots are presented here with full length blots presented in Fig. S12. (D) Quantification of western blot revealed a significant loss of FOXF1 in *αSMACreER;Foxf1^−/−^* livers. Quantification was averaged across four blots. FOXF1 levels were internally normalized to ACTIN for each sample. *P<0.05. (E) FOXF1 staining is detected in liver parenchyma and fibrotic regions (yellow arrows). FOXF1 staining is decreased in fibrotic regions of *αSMACreER;Foxf1^−/−^* livers (white arrows).

Since FOXF1 is expressed in HSCs in the liver (Kalinichenko et al., 2003), we examined the efficiency of *Foxf1* deletion in our experimental model, using immunostaining for FOXF1 and DES. Without CCl_4_ treatment, FOXF1 was observed in cell nuclei of DES-positive stellate cells in *Foxf1^fl/fl^* and *αSMACreER;Foxf1^fl/fl^* livers (Fig. 2A). After CCl_4_ and Tam treatment, FOXF1 staining was reduced in DES-positive cells of *αSMACreER;Foxf1^−/−^* livers but not in *Foxf1^fl/fl^* livers (Fig. 2A). We also immunostained liver sections for FOXF1 and αSMA, a marker of MFs (Rockey et al., 2013). While αSMA was not detected in parenchyma of quiescent livers, αSMA staining was increased after CCl_4_ injury. FOXF1 was detected in MFs of control livers but not in *αSMACreER;Foxf1*^−/−^ livers (Fig. 2B). Quantitative counts of FOXF1-expressing cells demonstrated that the number and percentage of FOXF1^+^ MFs (FOXF1^+^ αSMA^+^) were reduced whereas the number and percentage of FOXF1^−^ MFs (FOXF1^−^ αSMA^+^) were elevated in injured *αSMACreER;Foxf1^−/−^* livers compared to controls (Fig. S3). FOXF1 protein and mRNA were increased in CCl_4_-treated *Foxf1^fl/fl^* livers and purified HSCs (Fig. 2C,D; Fig. S3) but not in the *αSMACreER;Foxf1^−/−^* livers (Fig. 2C,D). The loss of FOXF1 in *αSMACreER;Foxf1^−/−^* livers occurred in periportal regions while pericentral regions were unaffected (Fig. 2E). The *αSMA-CreER* transgene allows for the maintained presence of FOXF1 for HSC activation (Kalinichenko et al., 2003) and only deletes FOXF1 after *αSMA* is expressed in MFs. Thus, *αSMA-CreER* transgene effectively deletes *Foxf1* from hepatic MFs during CCl_4_-mediated chronic liver injury.

### Deletion of *Foxf1* reduces MMP9 activity in CCl_4_-injured livers

**Fig. 3.**
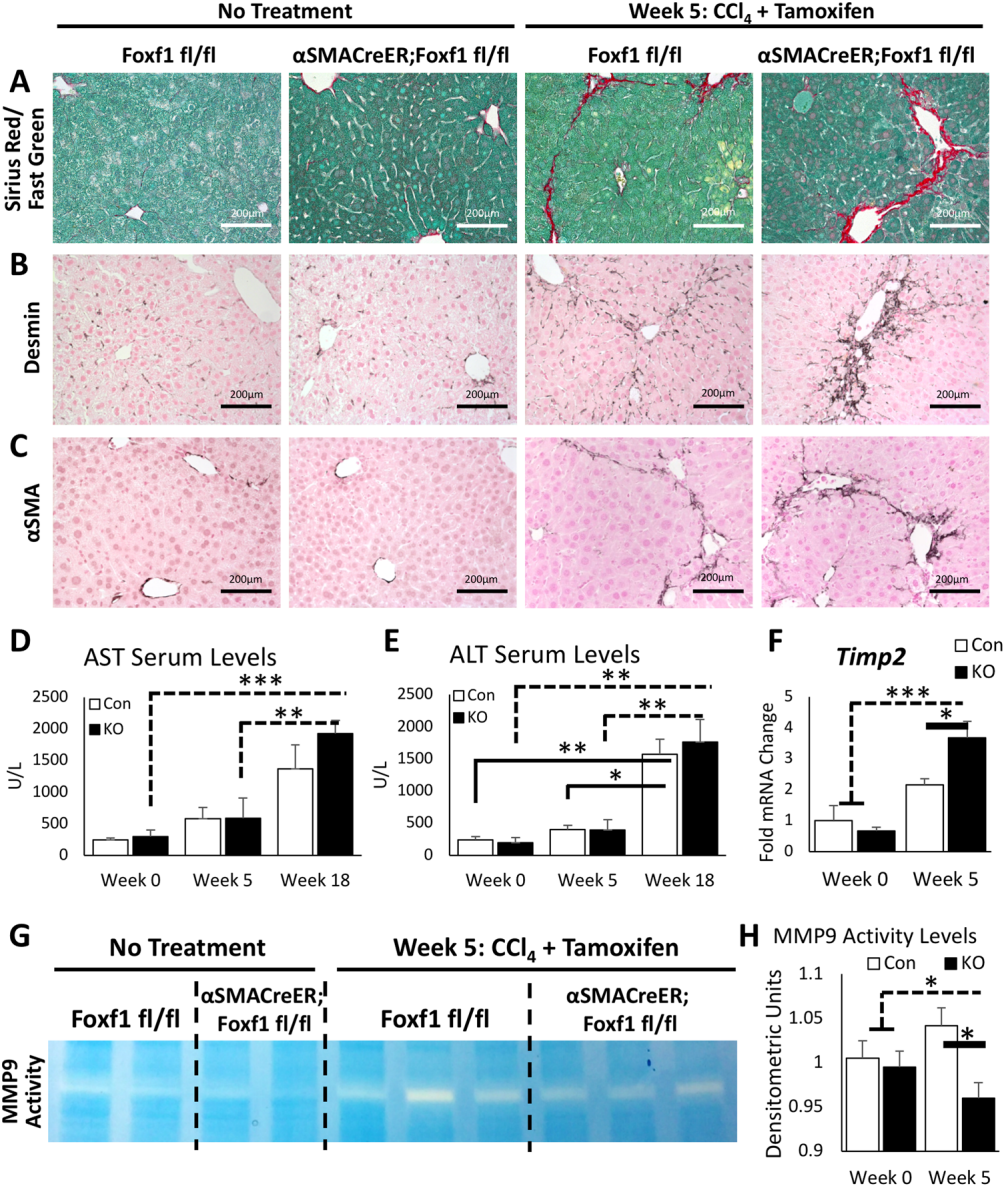
**Deletion of *Foxf1* from myofibroblasts increases liver fibrosis and inhibits MMP9 activity.** (A) Sirius Red/Fast Green staining demonstrates increased collagen deposition between portal triads in CCl_4_-treated *αSMACreER;Foxf1^−/−^* livers. (B,C) Immunohistochemistry shows increased staining for DES and αSMA in CCl_4_-treated *αSMACreER;Foxf1^−/−^* livers. (D,E) Serum enzymatic analysis demonstrates increased AST and ALT levels after chronic CCl_4_-induced liver injury. *Foxf1* deletion does not affect AST or ALT in blood serum. For AST levels: *n*=3 control mice and *n*=4 KO mice in week 0; *n*=5 control mice and *n*=5 KO mice in week 5; *n*=4 control mice and *n*=7 KO mice in week 18. For ALT levels: *n*=5 control mice and *n*=6 KO mice in week 0; *n*=7 control mice and *n*=8 KO mice in week 5; *n*=4 control mice and *n*=7 KO mice in week 18. (F) Increased *Timp2* mRNA in CCl_4_-treated *αSMACreER;Foxf1^−/−^* livers is found by qRT-PCR. (G) Representative zymography gel shows decreased MMP9 activity in CCl_4_-treated *αSMACreER;Foxf1^−/−^* livers. Cropped gel is presented here with full gel presented in Fig. S12. (H) Quantification of zymography gels reveals a significant decrease in MMP9 activity in CCl_4_-treated *αSMACreER;Foxf1^−/−^* livers. Quantification was averaged across three gels. *P<0.05, **P<0.01, ***P<0.001.

Histological staining with Sirius Red/Fast Green showed a significant increase in collagen accumulation in *αSMACreER;Foxf1^−/−^* livers after five weeks of CCl_4_ treatment (Fig. 3A; Fig. S4). Increased fibrosis in *Foxf1*-deficient livers was confirmed by immunostaining for DES and αSMA (Fig. 3B,C). To examine the consequences of extended CCl_4_ treatment, we treated mice with CCl_4_ for 18 weeks. While hepatic enzymes AST and ALT were increased in blood serum after 18 weeks of CCl_4_, there was no difference between CCl_4_ treated *αSMACreER;Foxf1^−/−^* and control mice (Fig. 3D,E). Blood serum protein (albumin, globulin) and bilirubin (direct, indirect) levels were not affected after deletion of *Foxf1* (Fig. S5). Collagen accumulation was time-dependent (Fig. S6), and after 18 weeks of CCl_4_ treatment, resulted in widespread liver fibrosis (Fig. S7) and in rare cases, the appearance of visible tumors (Fig. S7).

Since MMP9 plays an important role in collagen degradation after liver injury (Duarte et al., 2015), we evaluated mRNA expression of *Mmp9* and its inhibitor, *Timp2*, in liver tissue. *Timp2* mRNA was increased in CCl_4_-injured *αSMACreER;Foxf1^−/−^* livers compared to controls (Fig. 3F). Although *Mmp9* mRNA was unchanged (Fig. S8), evaluation of MMP9 activity through zymography demonstrated a significant decrease in enzymatic activity of MMP9 in *αSMACreER;Foxf1^−/−^* livers after CCl_4_ treatment (Fig. 3G,H). *Mmp8*, *Mmp13*, *Mmp16*, *Timp1* and *Timp3* mRNA levels were not affected in *Foxf1*-deficient livers (Fig. S8). Thus, *Foxf1* deletion from MFs increases *Timp2* mRNA and reduces MMP9 activity in fibrotic livers.

### Deletion of *Foxf1* does not influence cellular proliferation in fibrotic livers

**Fig. 4.**
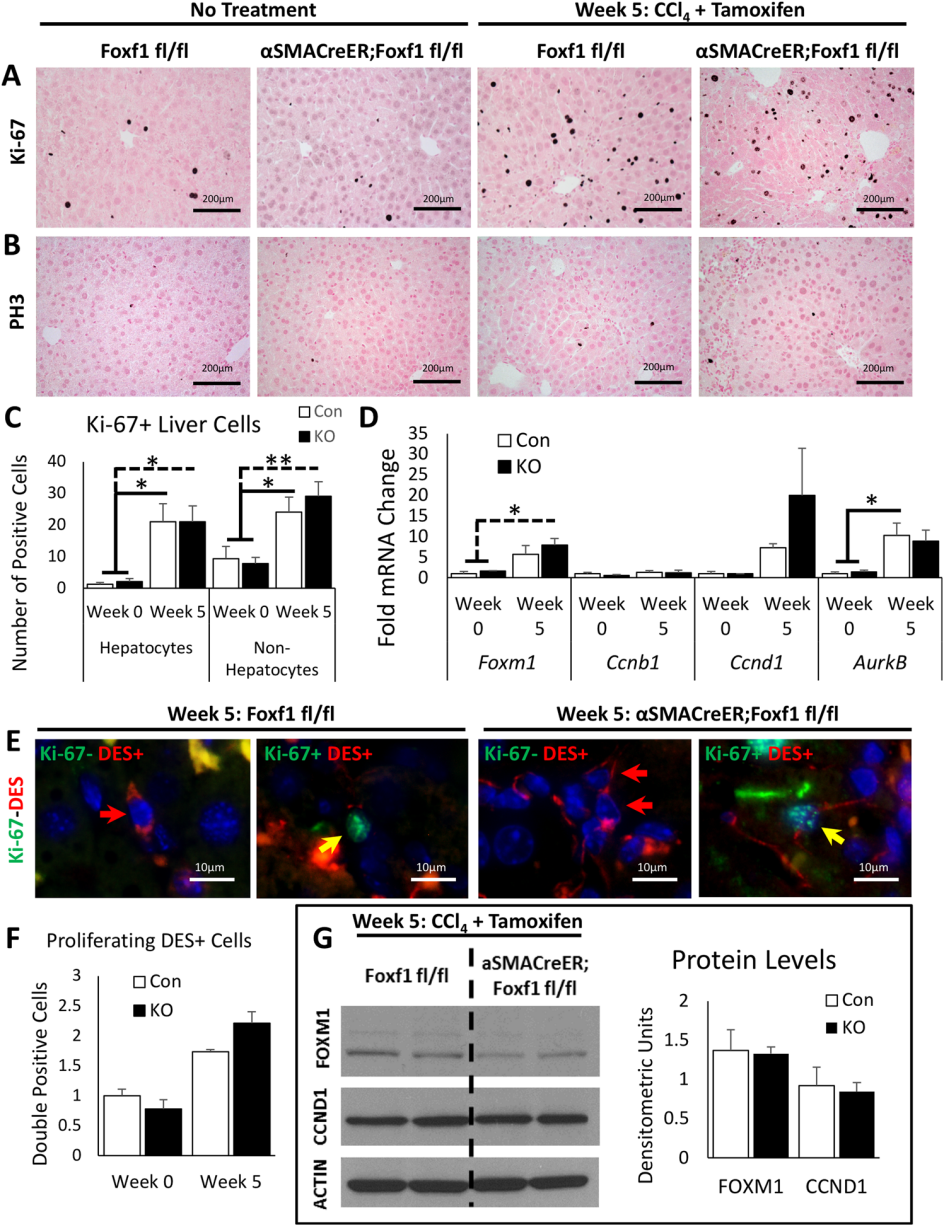
**Deletion of *Foxf1* does not influence proliferation of hepatic myofibroblasts.** (A) Ki-67 staining shows a significant increase in cell proliferation following CCl_4_-induced liver injury. No difference in Ki-67 staining is detected between *Foxf1^fl/fl^* and *αSMACreER;Foxf1^−/−^* livers. (B) PH3 staining shows no significant changes in mitotic rates between *Foxf1^fl/fl^* and *αSMACreER;Foxf1^−/−^* livers. (C) The number of Ki-67^+^ hepatocytes and non-hepatocytes in *Foxf1^fl/fl^* livers was similar to those in *αSMACreER;Foxf1^−/−^* livers. Numbers of Ki-67^+^ cells were counted in 20–25 random 200× microscope fields using *n*=3 mice per group in week 0 and *n*=7 control mice and *n*=6 KO mice in week 5. (D) qRT-PCR was used to measure mRNAs in whole liver RNA. mRNAs were normalized to *Actb*. *n*=3 mice per group in week 0; *n*=5 mice per group in week 5. (E) Co-localization of Ki-67 with DES shows the presence of Ki-67^+^ MFs in livers of CCl_4_-treated mice. (F) Quantification of co-localization of Ki-67 with DES shows no difference in the number of Ki-67^+^ DES^+^ cells in *Foxf1^fl/fl^* livers compared to *αSMACreER;Foxf1^−/−^* livers. (G) Western blot shows no significant difference in total liver protein levels of FOXM1 and CCND1 between *Foxf1^fl/fl^* and *αSMACreER;Foxf1^−/−^* livers. Cropped blots are presented here with full length blots presented in Fig. S12. *P<0.05, **P<0.01.

We evaluated proliferation markers to investigate if the increased fibrosis in *αSMACreER;Foxf1^−/−^* livers was due to an expansion of the stromal cells. While cellular proliferation was increased after CCl_4_ treatment, there were no significant differences in the number of proliferating hepatocytes or non-hepatocytes between *Foxf1^fl/fl^* and *αSMACreER;Foxf1^−/−^* livers (Fig. 4A–C; Fig. S9). Hepatocytes and non-hepatocytes were identified through distinct morphological appearances (Malarkey et al., 2005) from high magnification images. mRNAs of proliferation-specific genes *Foxm1*, *Ccnb1*, *Ccnd1*, and *AurKB (*Wang et al., 2009; Kalin et al., 2011*;* Ren et al., 2013*)* were unchanged between *Foxf1^fl/fl^* and *αSMACreER;Foxf1^−/−^* livers (Fig. 4D). Proliferating HSCs and MFs were detected in CCl_4_-treated livers by co-localization of Ki-67 with DES (Fig. 4E) and αSMA (Fig. S9); however, there were no changes in the number of Ki-67-positive HSCs and MFs after deletion of *Foxf1* (Fig. 4F). Protein levels of proliferation-specific genes FOXM1 and CCND1 were unaltered in *αSMACreER;Foxf1^−/−^* livers compared to controls (Fig. 4G). Thus, *Foxf1* deletion does not affect proliferation of HSCs and MFs after chronic CCl_4_ liver injury.

### RNA-seq analysis identified direct FOXF1 target genes critical for ECM deposition and hepatic fibrosis

**Fig. 5.**
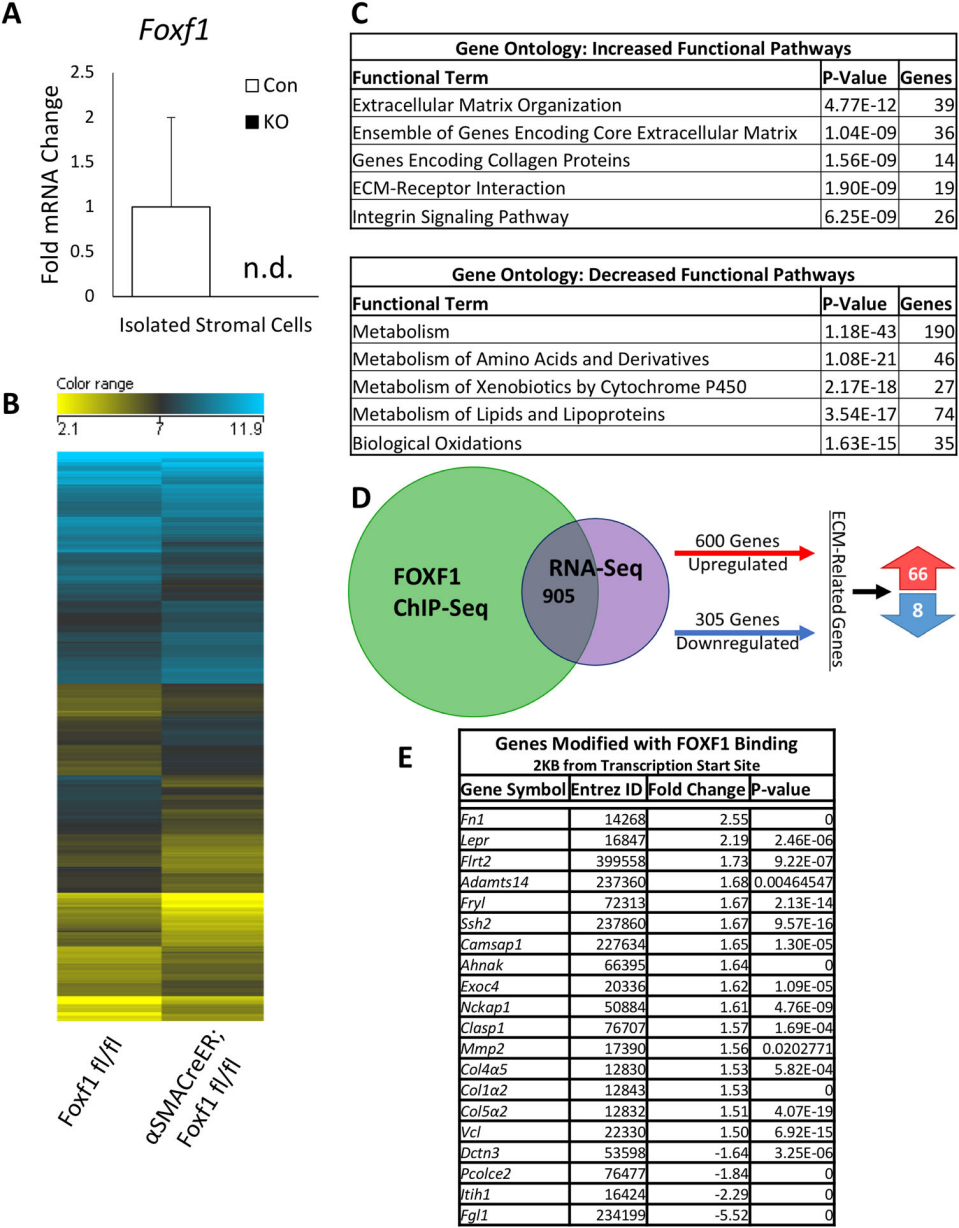
**FOXF1 deletion alters expression of pro-fibrotic genes in hepatic myofibroblasts.** (A) qRT-PCR analysis of primary hepatic stromal cells submitted for RNA sequencing shows that *Foxf1* mRNA is not detected (n.d.) in *αSMACreER;Foxf1^−/−^* livers. Samples were pooled for further analysis. (B) Heat map shows differentially expressed genes in stromal cells from *Foxf1^fl/fl^* and *αSMACreER;Foxf1^−/−^* livers after chronic CCl_4_-induced hepatic injury as identified by RNA-seq analysis. (C) Biological processes influenced by the deletion of *Foxf1* were identified using ToppFunn. *P*-values and number of genes are listed for each classification. (D) 905 overlapping genes were identified between ChIP-seq (GEO accession GSE100149) and RNA-seq (GEO accession GSE123726) data, of which 74 were ECM-related genes. (E) Table shows 20 ECM-related genes identified by ChIP-seq and RNA-seq (FOXF1 binding was analyzed within 2 KB from transcriptional start site).

In order to identify FOXF1 target genes, RNA-seq (GEO accession GSE123726) was performed on primary hepatic stromal cells (containing MFs and HSCs) isolated from CCl_4_-treated *Foxf1^fl/fl^* and *αSMACreER;Foxf1^−/−^* livers. Purified cells expressed *Des* and *Acta2*, but lacked hepatocyte (Nikoozad et al., 2014) and Kupffer cell (Yang et al., 2013*)* markers (Fig. S10). *Foxf1* mRNA was lost in isolated *αSMACreER;Foxf1^−/−^* stromal cells (Fig. 5A), a finding consistent with efficient deletion of *Foxf1* from CCl_4_-treated livers. The RNA-seq was used to compare differential gene expression patterns between the *Foxf1^fl/fl^* and *αSMACreER;Foxf1^−/−^* stromal cells. The differential gene expression in the two groups are represented in a heat map (Fig. 5B). Gene ontology demonstrated that increased functional pathways for the *αSMACreER;Foxf1^−/−^* mice were related to ECM regulation, while decreased functional pathways included normal liver functions and metabolism (Fig. 5C). RNA-seq analysis was cross referenced with FOXF1 ChIP-seq analysis (GEO accession GSE100149). 905 genes were common between RNA-seq and ChIP-seq (Fig. 5D), which include 74 genes related to ECM deposition and fibrosis. ChIP-seq proximity analysis revealed that 20 of these ECM genes had FOXF1 binding sites within 2KB of the transcription start site (Fig. 5E).

**Fig. 6.**
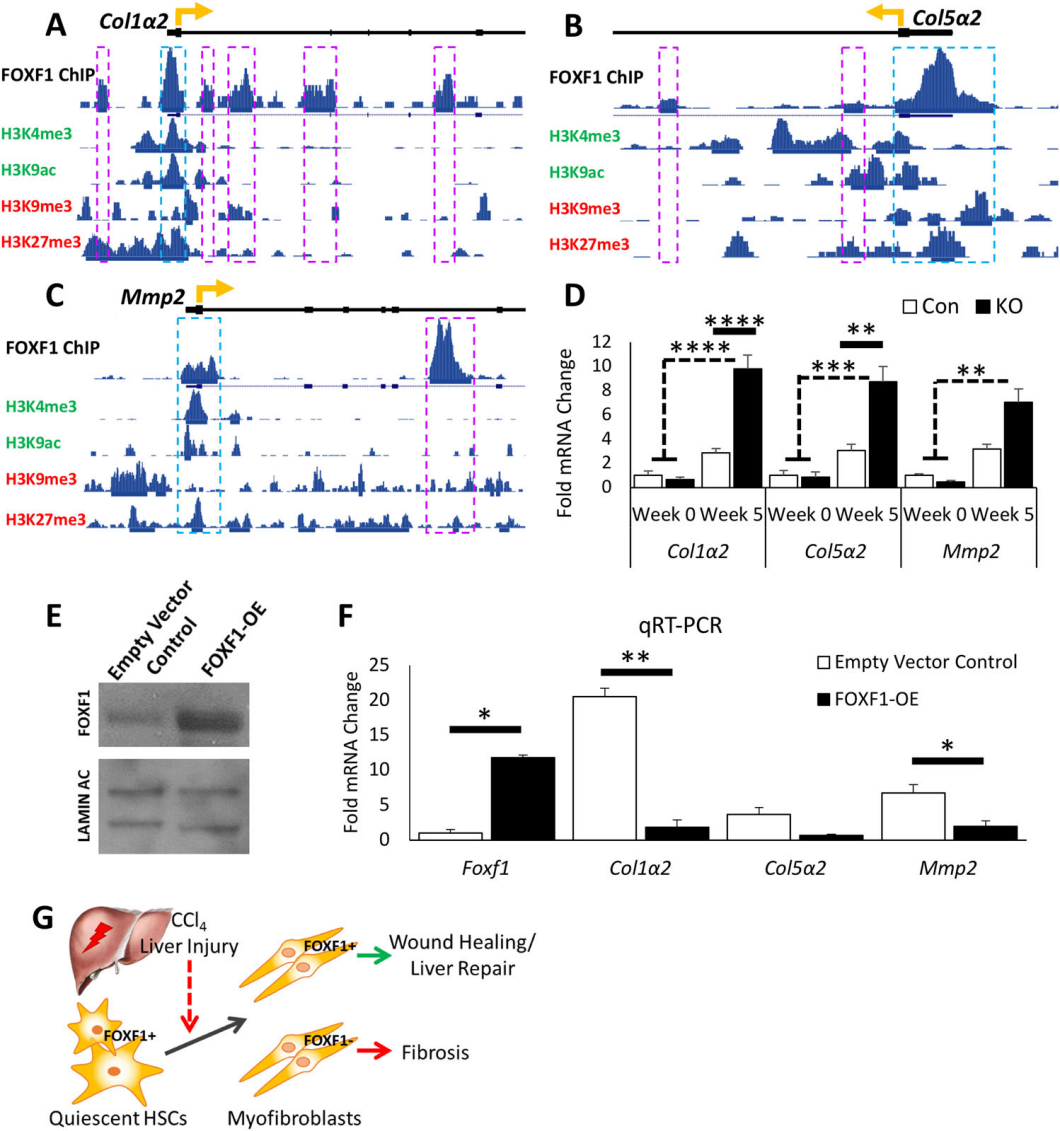
**FOXF1 binds to DNA regulatory regions of *Col1α2*, *Col5α2* and *Mmp2*.** (A–C) ChIP-seq shows FOXF1 binding near the transcriptional start sites in *Col1α2*, *Col5α2* and *Mmp2* gene loci. Histone modification marks of enhancers (H3K4me3, H3K9ac) and repressors (H3K9me3, H3K27me3) are aligned with FOXF1-binding regions. Significant areas of FOXF1 binding are marked with boxes, with blue boxes indicating the binding site is within gene promoter region. Gene transcriptional start sites are marked with a directional yellow arrow. (D) qRT-PCR analysis shows the significant increase of *Col1α2*, *Col5α2* and *Mmp2* mRNAs in the isolated stromal cells of CCl_4_-treated *αSMACreER;Foxf1^−/−^* livers. For *Col1α2* and *Col5α2*: *n*=3 mice per group in week 0; *n*=5 mice per group in week 5. For *Mmp2*: *n*=3 mice per group in week 0; *n*=6 control mice and *n*=4 KO mice in week 5. (E) Western blot shows increase in FOXF1 expression in isolated HSCs after FOXF1-overexpression. Cropped blots are presented here with full length blots presented in Fig. S12. (F) qRT-PCR shows an increase of *Foxf1* mRNA and a decrease of *Col1α2*, *Col5α2* and *Mmp2* mRNAs in isolated HSCs after FOXF1-overexpression. (G) Diagram of hepatic fibrosis in *Foxf1*-deficient mice shows that the loss of FOXF1 promotes ECM deposition and exacerbated fibrosis after CCl_4_-treatment. *P<0.05, **P<0.01, ***P<0.001, ****P<0.0001.

Interestingly, *Col1α2*, *Col5α2* and *Mmp2* were among the 20 ECM-related genes that had FOXF1 binding sites within the gene loci (Fig. S11, Table S1). COL1α2 and COL5α2 are common ECM components in fibrotic livers (Mak et al., 2016), whereas MMP2 is a collagenase that is increased during liver fibrosis and associated with disease progression (Benyon et al., 1996). Expression of *Col1α2*, *Col5α2* and *Mmp2* mRNAs were increased in CCl_4_-treated *αSMACreER;Foxf1^−/−^* livers as shown by RNA-seq and qRT-PCR (Fig. 5E, Fig. 6D), suggesting a negative regulation by FOXF1. The presence of gene silencing histone methylation marks H3K9me3 and H3K27me3 (Dong and Weng, 2013; Bernstein et al., 2006) in FOXF1-binding promoter regions (Fig. 6A–C) is consistent with negative regulation of these genes by FOXF1. In order to confirm the regulation of *Col1α2*, *Col5α2* and *Mmp2* by FOXF1, we overexpressed FOXF1 in isolated murine HSCs (Fig. 6E). Lentiviral-mediated overexpression of FOXF1 decreased *Col1α2* and *Mmp2 in vitro* (Fig. 6F). Thus, FOXF1 negatively regulates expression of pro-fibrotic genes in MFs. Altogether, FOXF1 expression in myofibroblasts is essential to inhibit liver fibrosis after chronic liver injury (Fig. 6G).

## DISCUSSION

Myofibroblast activation is a key mechanism in the development of hepatic fibrosis. However, transcriptional regulation of myofibroblasts during liver fibrogenesis remains poorly characterized. In the present study, we found that the deletion of *Foxf1* in MFs during chronic CCl_4_-mediated injury exacerbated liver fibrosis, increased collagen deposition and stimulated expression of pro-fibrotic genes. ECM-related proteins were identified as novel FOXF1 transcriptional targets, suggesting that FOXF1 plays an important role in the regulation of ECM and collagen deposition during the progression of hepatic fibrosis.

Previous studies have focused on the role of FOXF1 in acute liver injury using a single CCl_4_ administration to *Foxf1^+/−^* mice. These studies demonstrated that FOXF1 is necessary for HSC activation to promote liver repair (Kalinichenko et al., 2003). CCl_4_-treated *Foxf1^+/−^* mice exhibited diminished collagen depositions and increased mortality after the liver injury (Kalinichenko et al., 2003). A recently published model of *Foxf1*-silencing using a lipid-based nanoparticle system to deliver *Foxf1* siRNA to the liver demonstrated attenuated collagen deposition when *Foxf1* siRNA was delivered 48 h prior to bile duct ligation (Abshagen et al., 2015). It is likely that *Foxf1* siRNA inhibited FOXF1 signaling in hepatic stellate cells, decreasing their activation and subsequent collagen depositions into the liver tissue, a finding consistent with previous studies using *Foxf1^+/−^* mice (Kalinichenko et al., 2003). Recently, a model of chronic hepatic injury using CCl_4_-injections, similar to the present study, was unsuccessful in silencing *Foxf1* expression using the same lipid based system to deliver *Foxf1* siRNA (Abshagen et al., 2015, 2017). This method involved four weeks of IP CCl_4_-injections before two weeks of treatment with *Foxf1* siRNA (Abshagen et al., 2017). It is possible that the lack of *Foxf1* silencing was due to inability of nanoparticles to target hepatic MFs. In the current study, we utilized a conditional genetic mouse model to delete *Foxf1* in MFs during CCl_4_-induced hepatic fibrosis which shares multiple histological similarities with human disease (Masugi et al., 2018; Bataller and Brenner, 2005). Interestingly, the loss of *Foxf1* in MFs resulted in increased collagen deposition, causing severe fibrotic lesions between hepatic portal triads in *αSMACreER;Foxf1^−/−^* livers. Our studies suggest that FOXF1 inhibits production of collagen and ECM during the progression of liver fibrosis. Increased fibrosis in *Foxf1*-deficient mice was associated with the appearance of liver tumors, a finding consistent with increased tumor formation in patients with liver cirrhosis (EASL-EORTC et al., 2018). Our studies suggest that maintaining *Foxf1* expression can be beneficial in patients with advanced liver fibrosis to inhibit fibrotic responses and decrease the risk of liver tumorigenesis.

In the present study, collagens were significantly increased in *αSMACreER;Foxf1^−/−^* livers after chronic CCl_4_-treatment. Desmin and αSMA were both increased in *αSMACreER;Foxf1^−/−^* livers; however, there were no differences in the number of proliferating cells between *Foxf1^fl/fl^* and *αSMACreER;Foxf1^−/−^* livers. Previously, FOXF1 has been shown to stimulate cell proliferation in lung endothelial cells (Ren et al., 2014; Bolte et al., 2017) and in rhabdomyosarcoma tumor cells (Milewski et al., 2017). Surprisingly, we found that deletion of *Foxf1* from MFs does not affect their proliferation during liver fibrogenesis. It is possible that FOXF1 requires additional co-activator or co-repressor proteins (that are not present in MFs) to regulate cellular proliferation. Additionally, we found an increase in *Timp2* expression with a decrease in MMP9 activity in *αSMACreER;Foxf1^−/−^* livers. Since it is well-known that TIMPs and MMPs regulate ECM depositions to balance the scaring and healing processes during fibrosis (Duarte et al., 2015), it is possible that the loss of *Foxf1* alters the TIMP/MMP balance to allow accumulation of collagens without the degradation mechanisms necessary for proper wound healing. Interestingly, MMP9 has been implicated in HSC to MF transdifferentiation (Han et al., 2007) in addition to its roles in ECM degradation (Duarte et al., 2015; Kurzepa et al., 2014). Therefore, decreased MMP9 activity can contribute to increased liver fibrosis in *αSMACreER;Foxf1^−/−^* mice. Surprisingly, FOXF1 was increased in activated HSCs compared to quiescent HSCs. It is possible that FOXF1 is differentially regulated in HSCs compared to hepatic MFs, and that after liver injury, FOXF1 protects HSCs from differentiating into MFs through transcriptional repression of profibrotic genes.

Consistent with increased fibrosis in *Foxf1*-deficient livers, RNA-seq analysis revealed increased ECM-related functional pathways in a purified stromal cell population. Comparison with FOXF1 ChIP-seq data revealed 20 novel transcriptional targets of FOXF1, which include *Col1α2*, *Col5α2* and *Mmp2*, expression of which was increased in *Foxf1*-deficient cells.

COL1α2 is one of the most abundant ECM components in the liver along with COL1α1 and COL3α1 (Lai et al., 2011). COL5α2 is highly expressed with Collagens 1 and 3 and is important in regulating the assembly and structure of these collagens in the fibrotic matrix (Moriya et al., 2011). MMP2 acts as a collagenase, known to be activated during hepatic fibrosis (Benyon et al., 1996). In addition to increased mRNA levels of the genes in FOXF1-deficient cells, we found multiple FOXF1 binding sites within their gene promoter region and introns, suggesting direct transcriptional repression. This hypothesis is supported by the presence of H3K4me3 and H3K9ac, histone modifications associated with transcriptional repression (Dong and Weng, 2013; Rea et al., 2000), at FOXF1 binding sites. In summary, we have developed a novel genetic mouse model to study the role of FOXF1 in MFs during chronic liver injury. Using this model, we demonstrated that *Foxf1* expression in MFs is necessary to inhibit hepatic fibrosis and maintain the balance of collagen depositions, through transcriptional repression of pro-fibrotic genes.

## MATERIALS AND METHODS

### Mice

The *Foxf1^fl/fl^* mouse line was previously generated and bred into the C57Bl/6 mouse background (Ren et al., 2014; Cai et al., 2016). *Foxf1^fl/fl^* mice were bred with *αSMA-CreER* mice (Jackson Laboratory, 029925; Wendling et al., 2009) to generate *αSMACreER;Foxf1^fl/fl^* mice (Black et al., 2018). *αSMACreER;Foxf1^fl/fl^* mice were bred with *Foxf1^fl/fl^* mice and male pups were genotyped and used for all experiments at the age of 6–8 weeks. The following primers were used for genotyping: *αSMA-CreER* sense: 5′ TGCAACGAGTGATGAGGTTCGC 3′ and anti-sense: 5′ GATCCTGGCAATTTCGGCTATACG 3′; *αSMA-WT* sense 5′ GGTTTCTATTGCTACCAAGAGACAT 3′ and anti-sense: 5′ TGCACCAAACCCTGGACTAAGCAT 3′; *Foxf1^fl/fl^* sense: 5′ GCTTTGTCTCCAAGCGCTGC 3′ and anti-sense: 5′ TTCAGATCTGAGAGTGGCAGCTTC 3′. *Foxf1^fl/fl^* littermates were used as controls. To activate the conditional *Foxf1* knockout, tamoxifen (Tam) was given via intraperitoneal injection (40 mg/kg of body weight; Sigma-Aldrich) three days in a row at the beginning of each week starting at week 2 over the course of the chronic liver injury period. Hepatic injury was induced by intraperitoneal injections of carbon tetrachloride (CCl_4_; 1 μl/g of body weight 20% v/v; Sigma-Aldrich; diluted in sunflower seed oil) three times a week every other day over the course of the chronic liver injury period. The levels of aminotransferases AST and ALT, proteins albumin and globulin, and direct and indirect bilirubin were determined by serological analysis of blood serum as previously described (Sun et al., 2017; Ren et al., 2010). All animal studies were approved by the Institutional Animal Care and Use Committee (IACUC) of Cincinnati Children's Research Foundation and the NIH IACUC Guidebook. All experiments were covered under our animal protocol (IACUC2016-0038). The Cincinnati Children's Research Foundation Institutional Animal Care and Use Committee is an AAALAC and NIH accredited institution (NIH Insurance #8310801).

### Histology and immunohistochemistry

Paraffin-embedded liver sections were used for H&E staining, immunohistochemistry (IHC), or immunofluorescence (IF) as previously described (Ren et al., 2010; Kalinichenko et al., 2003; Wang et al., 2003). The following antibodies were used for immunostaining: FOXF1 (1:1000 IHC, 1:200 IF, R&D Systems), DES (1:500 IHC, 1:100 IF; Santa Cruz Technologies), αSMA (1:10,000 IHC, 1:5000 IF; Sigma-Aldrich), Ki-67 (1:1000 IHC, 1:200 IF; Thermo Fisher Scientific), Ki-67 (1:200 IF; BD Biosciences), and PH3 (1:10,000 IHC; Santa Cruz Technologies). Antibody-antigen complexes were detected using biotinylated secondary antibodies followed by avidin-biotin-horseradish peroxidase complex and 3,3′diaminobenzidine substrate (Vector Labs) as previously described (Kalinichenko et al., 2003; Ren et al., 2010; Wang et al., 2012). Sections were counterstained with Nuclear Fast Red (Vector Labs). For immunofluorescence imaging, secondary antibodies conjugated with Alexa Fluor 488 or Alexa Fluor 594 (Invitrogen/Molecular Probes) were used as described (Ustiyan et al., 2012; Wang et al., 2010). Cell nuclei were counterstained with DAPI (Vector Labs). Masson's Trichrome (BD Biosciences) and Sirius Red/Fast Green (Chondrex, Inc.) specialty stains were performed according to the manufacturer’s protocols. Brightfield images were obtained using a Zeiss AxioImage.A2 microscope. Fluorescent images were obtained using a Zeiss AxioPlan 2 microscope.

### qRT-PCR, western blot, and zymography

The caudate lobe of the liver was halved and used for RNA and protein studies. RNA was isolated using RNA Stat-60 (Tel-Test, Inc.) according to the manufacturer’s protocol and was reverse transcribed using the High Capacity Reverse Transcription Kit (Applied Biosystems) according to the manufacturer’s protocol. mRNAs of specific genes were measured by qRT-PCR using TaqMan probes (Applied Biosystems; Table S2) and the StepOnePlus Real-Time PCR system (Applied Biosystems) as described (Bolte et al., 2011, 2015, 2012; Wang et al., 2010). mRNAs were normalized to *Actb*. Protein extracts were isolated using cell lysis buffer as previously described (Pradhan et al., 2016) and used for either western blot analysis with Pierce ECL western blotting substrate (Thermo Fisher Scientific) or gel zymography (NOVEX) according to the manufacturer’s protocols. The following antibodies were used for protein blots: FOXF1 (1:1000, R&D Systems) (Bolte et al., 2017; Black et al., 2018; Ren et al., 2014), ACTIN (1:2000; Santa Cruz Biotechnology) (Pradhan et al., 2016), FOXM1 (1:3000; Santa Cruz Biotechnology) (Pradhan et al., 2016; Bolte et al., 2011), CCND1 (1:1000; Cell Signaling Technology) (Milewski et al., 2017). Protein band intensities were determined by ImageJ software and were normalized to ACTIN.

### Hepatic stellate cell isolation, transfection

Hepatic stellate cells were isolated from male C57Bl/6-WT mice (40–50 g), purified using Nycodenz gradient, and cultured as previously described (Pradhan et al., 2016; Dangi et al., 2012; Sumpter et al., 2012). Quiescent HSCs were harvested at day two after cell culture (Reinehr et al., 1998). After ten days in culture, activated MFs were harvested (Reinehr et al., 1998). The pMIEG3 bicistronic retroviral vector was used for FOXF1 protein overexpression as previously described (Pradhan et al., 2016). The cells were transfected as previously described (Singh et al., 2008). mRNAs in isolated MFs were normalized to *18 s* (Eukaryotic 18S rRNA Endogenous Control; Applied Biosciences). Protein in isolated MFs were normalized to LAMIN AC (1:10,000; Santa Cruz Biotechnology) (Pradhan et al., 2016).

### RNA sequencing

RNA was isolated from HSC/MF population purified from CCl_4_-treated *Foxf1^fl/fl^* and *αSMACreER;Foxf1^−/−^* livers using a differential plating method (Giassetti et al., 2016) that we modified. Briefly, liver cell suspension was plated on tissue culture dishes (Corning) and incubated for 2 h at 37°C. Supernatant and non-adherent cells were washed off and the adherent cell population was collected for experiments. Samples were pooled to generate the libraries using the TruSeq RNA library preparation kit and were sequenced on an Illumina HiSeq 2000 sequencer, generating approximately 10 M high quality single end reads (75 base-long reads). Alignment was performed using the Tophat/Cufflink pipeline (Trapnell et al., 2009, 2010). Finally, cuffmerge tool was used to generate Binary Alignment/Map files (BAM files) (Roberts et al., 2011). BAM files of RNA-seq data were analyzed using Avadis^®^ NGS Version 1.3.0 software. Reads were filtered to remove: (1) duplicate reads, (2) non-primary-matched reads, and (3) reads with alignment scores <95. Quantification was performed on the filtered reads against the RefSeq annotation. Data normalization was performed with the DESeq package. The sequencing depth was estimated by the read count of the gene with the median read count ratio across all genes. The method was based on the negative binomial distribution, which allows for less restrictive variance parameter assumptions than does the Poisson distribution. The false discovery rate was calculated according to the Benjamini and Hochberg algorithm (Benjamini and Hochberg, 1995). Genes with expression altered by a factor of 1.5 and a false discovery rate of 0.05 in *Foxf1^fl/fl^* cells compared with *αSMACreER;Foxf1^−/−^* cells were selected for gene set enrichment analysis using ToppGene Suite. Hierarchical clustering was performed by Ward's method using Euclidean distance metric. RNA-seq data are available at GEO accession GSE123726. RNA-seq data were compared to previously published ChIP-seq data (GEO accession GSE100149) using a two-way Venn diagram.

### Statistical analysis

Statistical significance differences in measured variables between control and experimental groups were assessed with a Student's *t-*test (two-tailed) or one-way analysis of variance (ANOVA) with Bonferroni post hoc test as appropriate. *P*<0.05 was considered to be significant, with *P*<0.05 indicated with *, *P*<0.01 indicated with **, *P*<0.001 indicated with ***, and *P*<0.0001 indicated with ****. Values for all measurements were expressed as mean±s.e. of mean.

## Supplementary Material

Supplementary information
