# Supplementary material for: The Forkhead box F1 transcription factor inhibits collagen deposition and accumulation of myofibroblasts during liver fibrosis
Source: Biol Open. 2019 Jan 23;8(2):bio039800. doi: 10.1242/bio.039800 (PMC6398469; doi:10.1242/bio.039800)
Supplement: Supplementary information [file biolopen-8-039800-s1.pdf]

Gene	Chromosome	TSS	FOXF1 Binding Site	
Col1a2	6	4,505,910	-1331	-1182
			-294	57
			368	524
			810	1167
			2017	2499
			4134	4416
			22703	22845
			31422	31862
			32255	32529
			32776	31022
Col5a2	1	45,502,820	-816	33
			304	472
			1904	2052
			7190	7332
			9270	9637
			10489	10681
			12506	12937
			14086	14369
			55083	55225
			82266	82514
			122097	122457
Mmp2	8	92,827,778	-573	439
			6349	7508
			19004	20139
			21290	21848

Table S1. FOXF1 binding sites as identified by ChIP-seq relative to the transcriptional start site.

Biology Open • Supplementary information

Gene Name	Assay Number
Actb	Mm00607939_s1
Acta2	Mm00725412_s1
Alb	Mm00802090_m1
AurkB	Mm01718146_g1
Cdkn1a	Mm01303209_m1
Cdkn1b	Mm00438168_m1
Ccnb1	Mm00838401_m1
Ccnd1	Mm00432359_m1
Clec4f	Mm00443934_m1
Col1a1	Mm00801666_g1
Col1a2	Mm00483888_m1
Col3a1	Mm00802331_m1
Col5a2	Mm00483675_m1
Des	Mm00802455_m1
Foxf1	Mm00487497_m1
Foxm1	Mm00514924_m1
Mmp13	Mm00624354_m1
Mmp16	Mm01210646_m1
Mmp2	Mm00439498_m1
Mmp8	Mm00439509_m1
Mmp9	Mm00442991_m1
Timp1	Mm00441818_m1
Timp2	Mm00441825_m1
Timp3	Mm00441826_m1

Table S2. List of TaqMan probes used in qRT-PCR analysis.

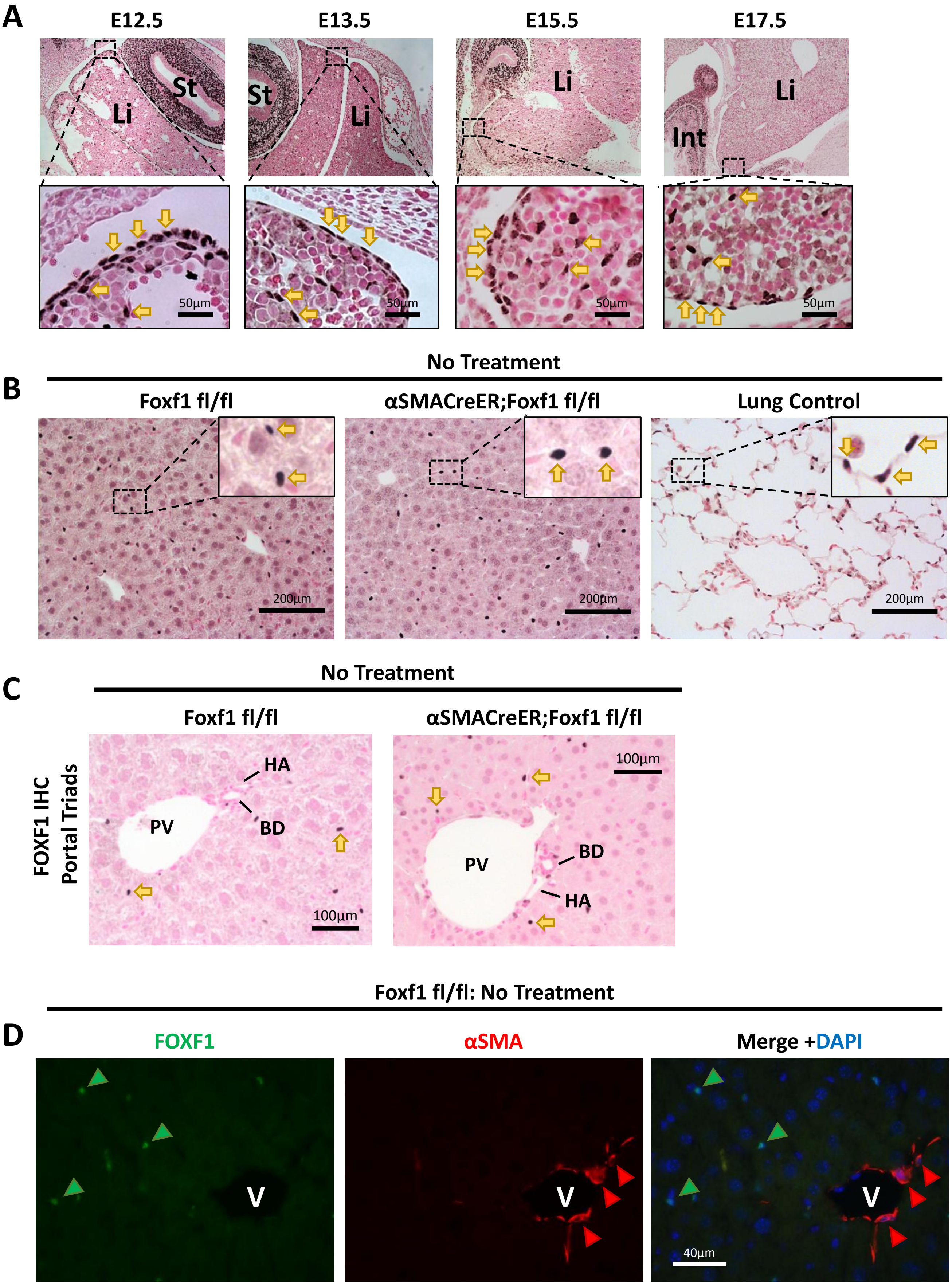

Biology Open • Supplementary information

*Figure S1. FOXF1 expression in mouse liver*s. (A) Immunostaining shows FOXF1 protein (dark brown) in nuclei of mesenchymal cells surrounding stomach (st) and intestine (int) of e12.5-17.5 mouse embryos. FOXF1 (yellow arrow) is also detected in mesothelial and stellate cells of the liver (li). Slides were counterstained with nuclear fast red (red). (B) Immunostaining shows FOXF1 (yellow arrow) expression in HSCs of uninjured livers. Lung sections were used as positive control for FOXF1 staining. (C) Immunostaining shows that FOXF1 (yellow arrow) is expressed in liver parenchyme but not in endothelial cells lining the portal vein (PV) and hepatic artery (HA). Bile ducts are shown as BD. (D) Co-localization of FOXF1 (green arrowheads) with αSMA (red arrowheads) shows that FOXF1 is not expressed in smooth muscle cells surrounding the portal vein (V) in uninjured *Foxf1*$^{fl/fl}$ livers.

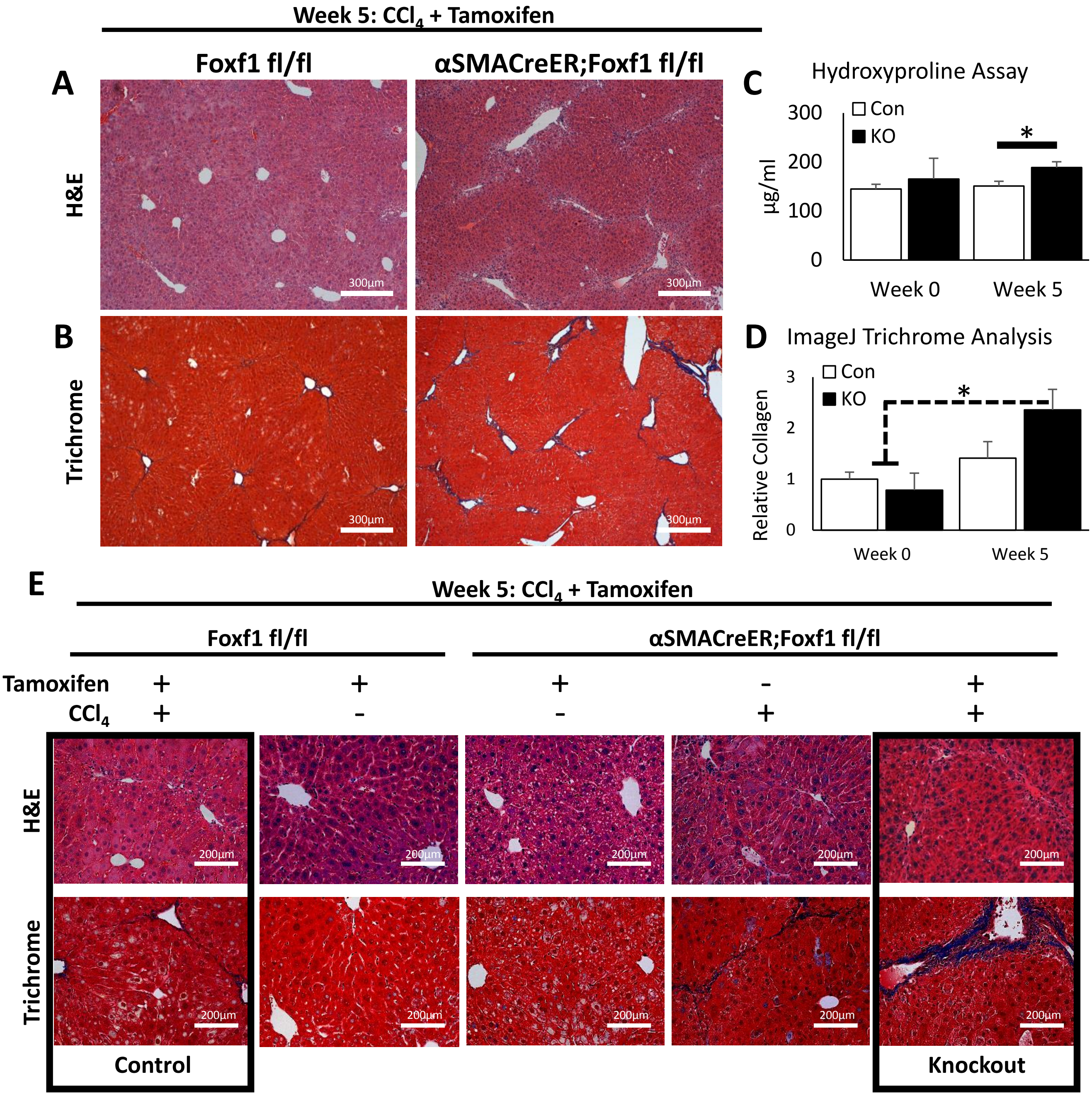

Figure S2. Treatment with tamoxifen alone does not induce hepatic fibrosis. (A) H&E and (B) Masson's trichrome staining of *Foxf1*$^{fl/fl}$ and *αSMACreER;Foxf1*$^{fl/fl}$ livers after 5-weeks of CCl_4 and Tam treatment show widespread hepatic fibrosis. (C) Collagen deposition was quantitated using the Hydroxyproline assay. n=2 mice per group in week 0; n=4 mice per group in week 5. (D) ImageJ analysis of Masson's trichrome images shows a significant increase in collagen in livers from *αSMACreER;Foxf1*$^{-/-}$ mice. n=3 mice per group in week 0; n=5 mice per group in week 5. (E) H&E and Masson's trichrome staining of *Foxf1*$^{fl/fl}$ and *αSMACreER;Foxf1*$^{fl/fl}$ livers from mice treated with Tam alone or in combination with CCl_4. Tam treatment does not induce hepatic fibrosis in either *Foxf1*$^{fl/fl}$ or *αSMACreER;Foxf1*$^{fl/fl}$ mice. Tam-mediated deletion of *Foxf1* exacerbates the fibrotic phenotype observed in CCl_4-treated *αSMACreER;Foxf1*$^{-/-}$ livers. P<0.05 is indicated with *.

Biology Open • Supplementary information

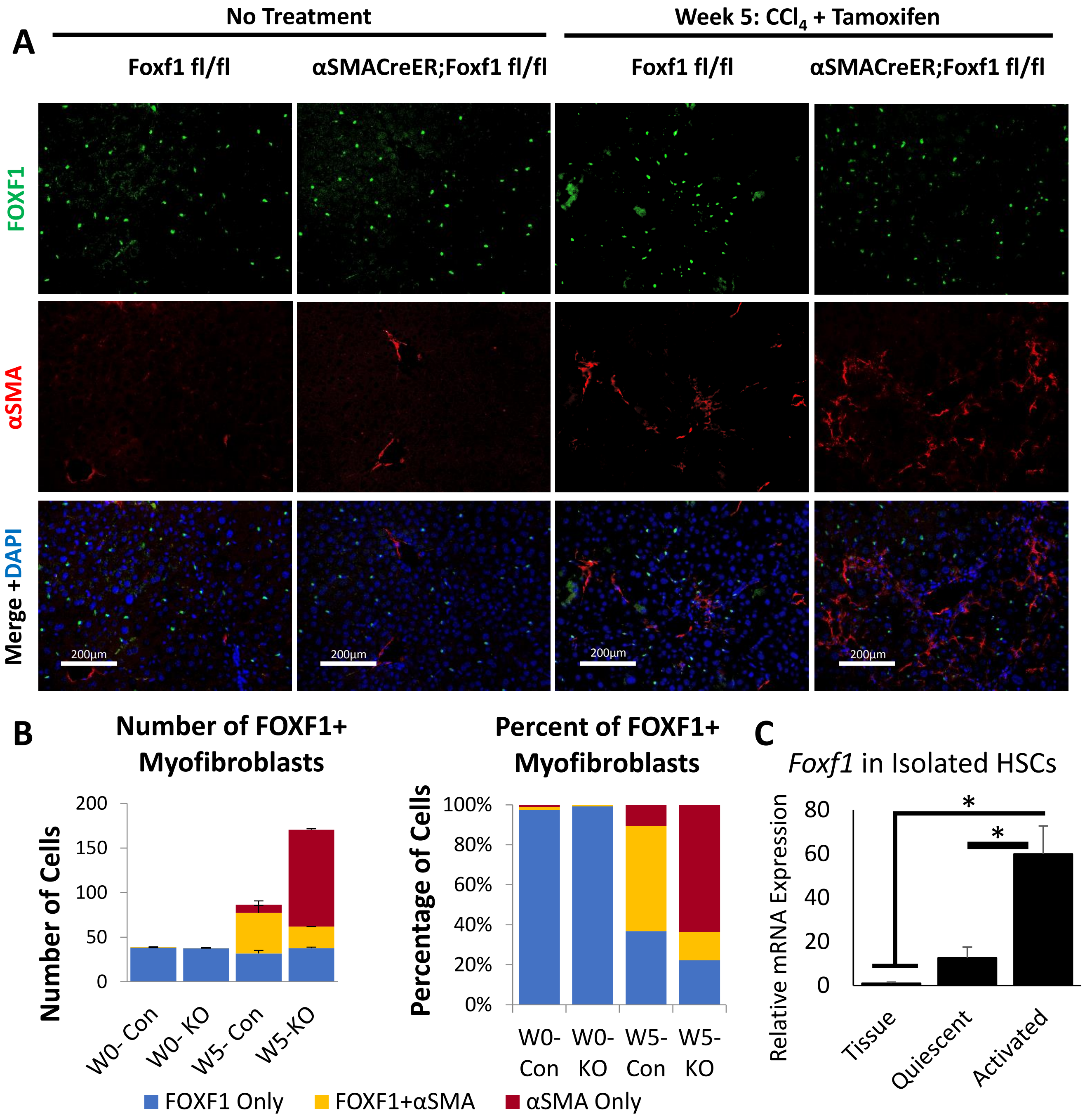

Figure S3. Number and percentage of FOXF1+ myofibroblasts are reduced in αSMACreER;Foxf1$^{-/-}$ livers. (A) Co-localization of FOXF1 with αSMA in *Foxf1$^{fl/fl}$* and *αSMACreER;Foxf1$^{-/-}$* livers. Scale bars 200 μm. (B) Counts of cells double stained for FOXF1 and αSMA show an increased number and percentage of αSMA+ FOXF1- MFs in CCl_4-treated *αSMACreER;Foxf1$^{-/-}$* livers compared to controls (W0 is Week 0; W5 is Week 5). (C) qRT-PCR shows a significant increase in *Foxf1* mRNA in HSCs isolated from C57Bl/6-WT mice at the activated stage compared to uninjured livers.

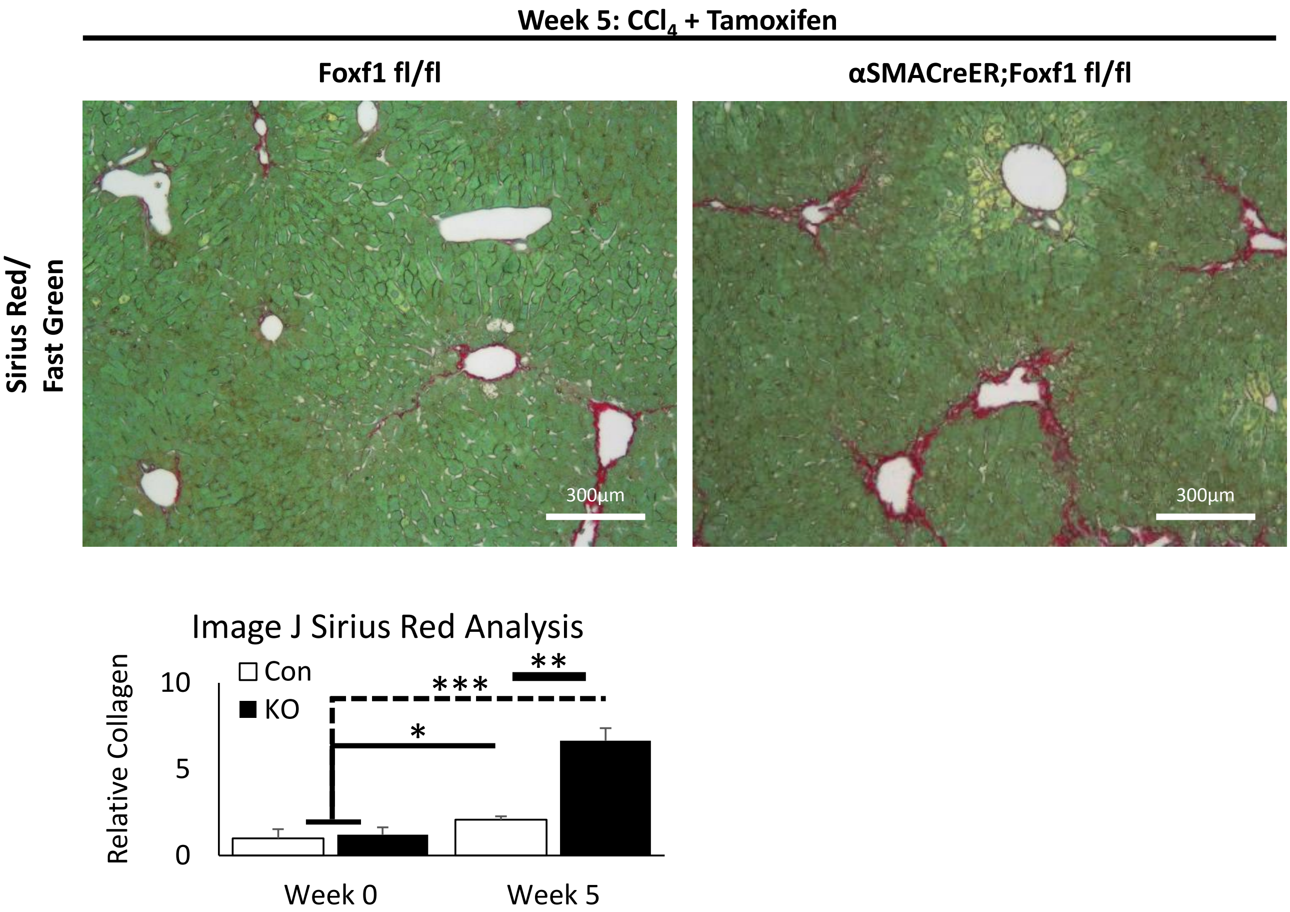

Figure S4. Increased collagen deposition in Foxf1-*deficient livers.* Sirius Red/Fast Green staining shows widespread collagen accumulation in *αSMACreER;Foxf1*$^{-/-}$ livers compared to *Foxf1*$^{fl/fl}$ livers. ImageJ analysis of Sirius Red/Fast Green images shows a significant increase in collagen in livers from *αSMACreER;Foxf1*$^{-/-}$ mice. n=3 mice for Con and n=4 mice for KO in week 0; n=4 mice for Con and n=5 mice for KO in week 5. P<0.05 is indicated with *, P<0.01 is indicated with **, P<0.001 is indicated with ***.

Biology Open • Supplementary information

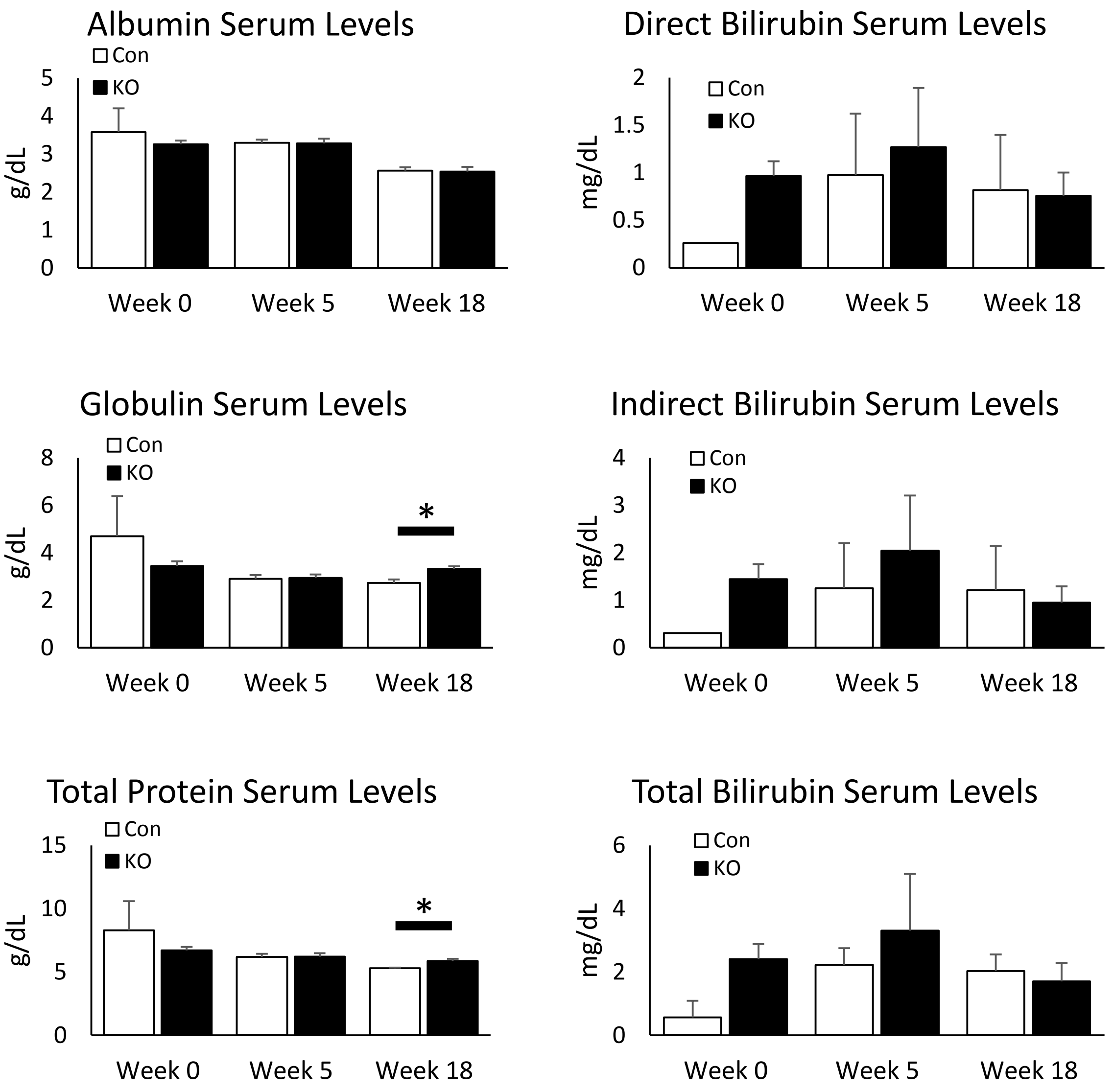

Figure S5. Deletion of Foxf1 *had no effect on serum protein or bilirubin levels.* The loss of *Foxf1* did not change serum albumin levels between control and *Foxf1*-deficient mice at 0, 5, or 18 weeks of CCl_4-treatment. Globulin levels were significantly increased at week 18 between *Foxf1*$^{fl/fl}$ and *αSMACreER;Foxf1*$^{-/-}$ livers, as were total protein levels. n=5 mice per group in weeks 0 and 5; n=3 control mice and n=5 KO mice in week 18. Deletion of *Foxf1* had no effect on bilirubin levels in blood serum. Data are shown as mean ± SEM.

Biology Open • Supplementary information

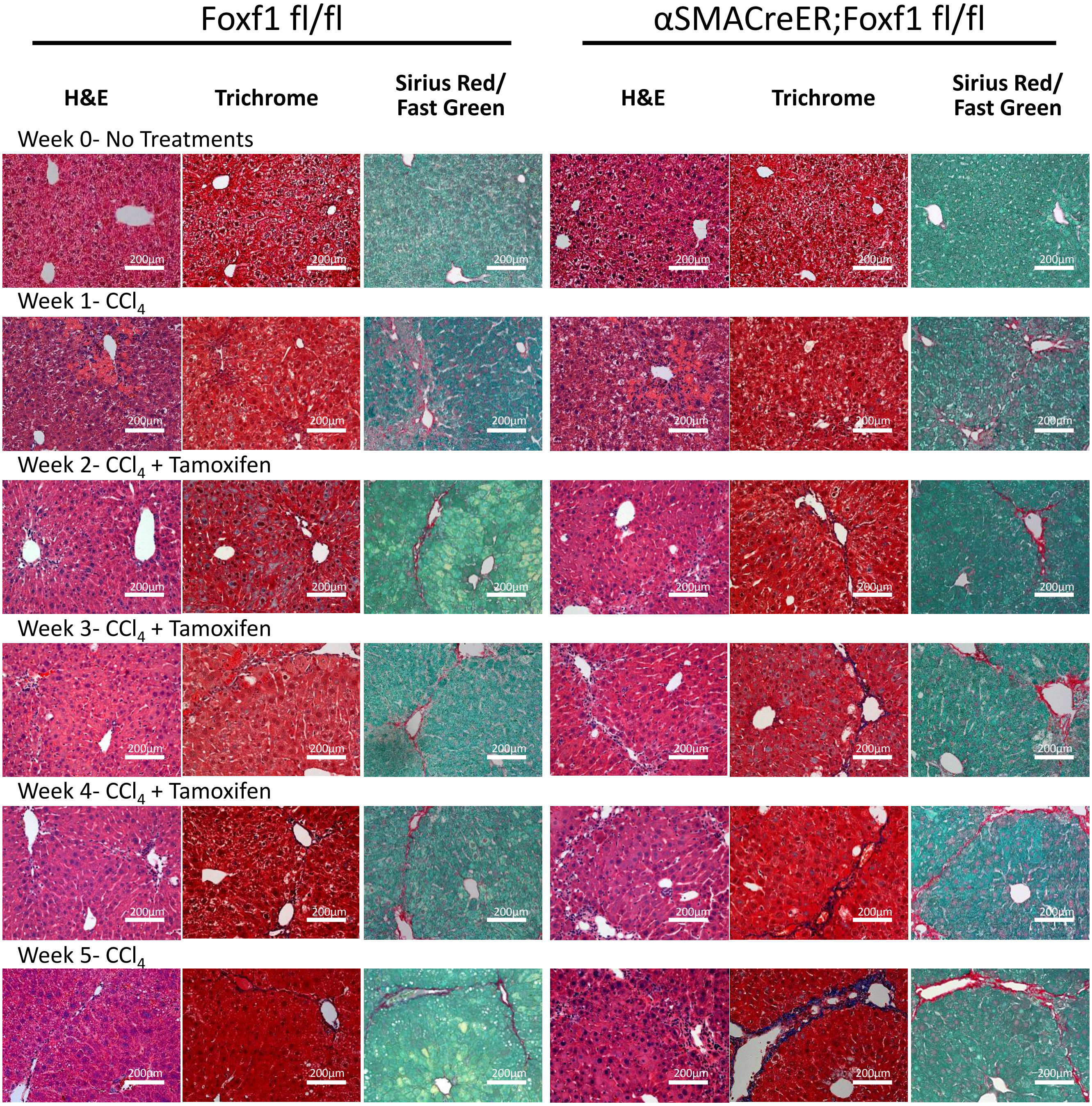

Figure S6. Collagen accumulation in Foxf1*-deficient livers is time-dependent.* H&E, Masson's trichrome, and Sirius Red/Fast green staining show a timecourse of CCl_4-induced hepatic fibrosis. Collagen depositions were greater in CCl_4-treated *αSMACreER;Foxf1*$^{-/-}$ livers.

Biology Open • Supplementary information

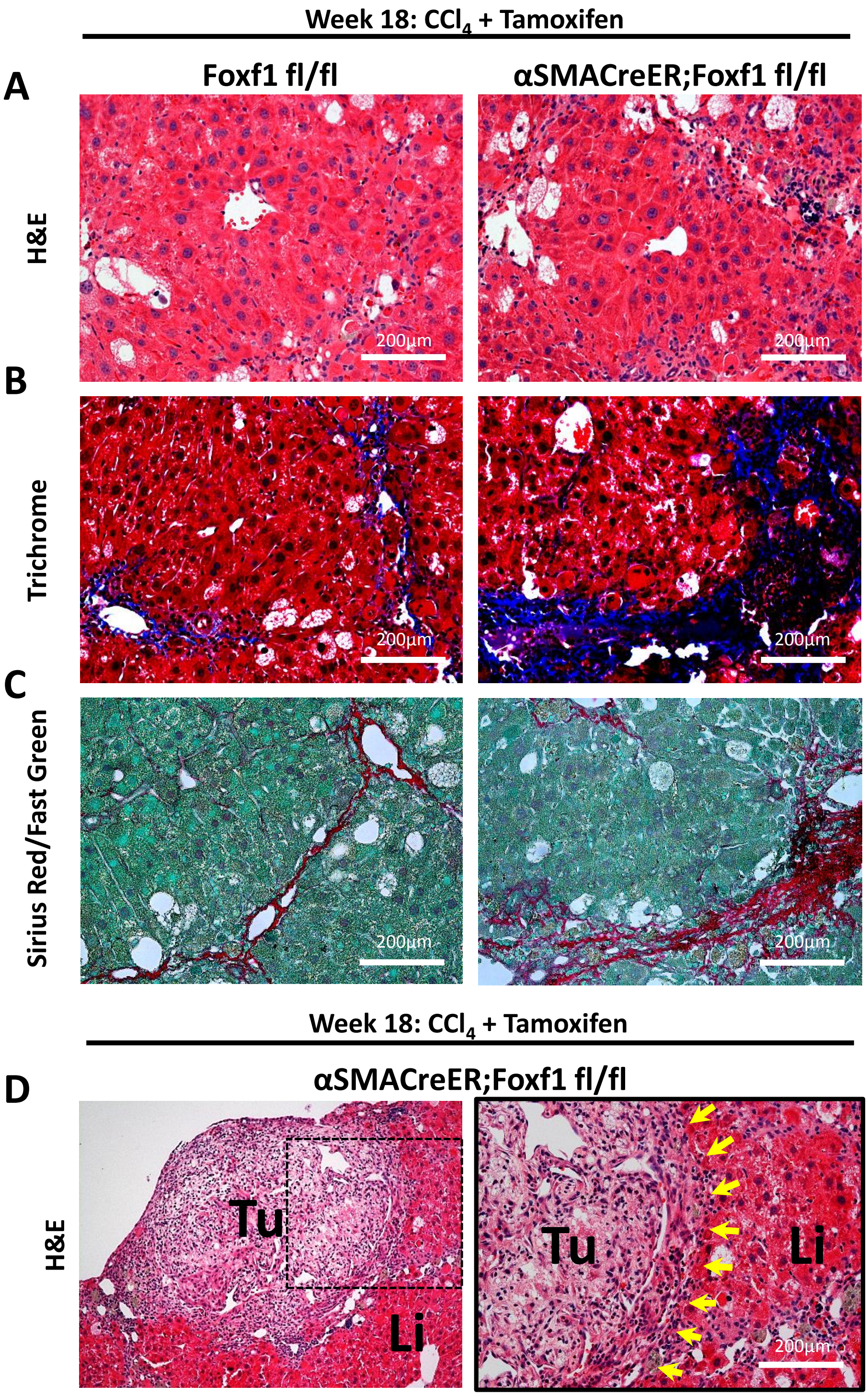

Figure S7. Widespread hepatic fibrosis and appearance of liver tumor in αSMACreER;Foxf1$^{-/-}$ *mouse after 18-weeks of* CCl_4 *treatment*. Representative liver sections stained for (A) H&E, (B) Masson's Trichrome, and (C) Sirius Red/Fast Green demonstrate that severe fibrotic lesions occur in *αSMACreER;Foxf1*$^{-/-}$ livers after 18-weeks of chronic hepatic injury. (D) H&E images show a hepatic tumor (Tu) in an *αSMACreER;Foxf1*$^{-/-}$ liver (Li) after 18-weeks of CCl_4-treatment. Tumors were found in 14.29% of mice (1 mouse out of n=7). Tumor boundaries are shown with arrows.

Biology Open • Supplementary information

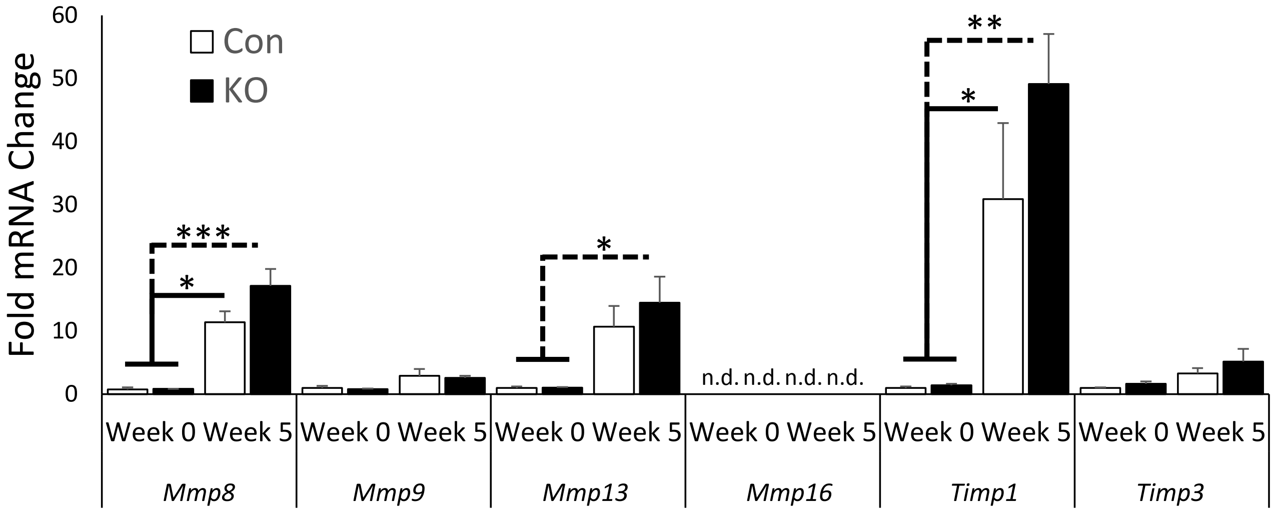

Figure S8. Deletion of Foxf1 *does not affect* Mmp8, Mmp9, Mmp13, Mmp16, Timp1, *or* Timp3 *mRNAs in* CCl_4*-treated livers*. qRT-PCR was used to measure mRNAs in whole liver extract. *Mmp16* was not detected (n.d.) in any sample tested. mRNAs were normalized to *Actb*. For *Mmp8*, *Mmp9*, *Mmp13*, *Mmp16*, *Timp1*, and *Timp3*: n=3 mice per group in week 0; n=5 mice per group in week 5. $P<0.05$ is indicated with *, $P<0.01$ is indicated with **, $P<0.001$ is indicated with ***.

Biology Open • Supplementary information

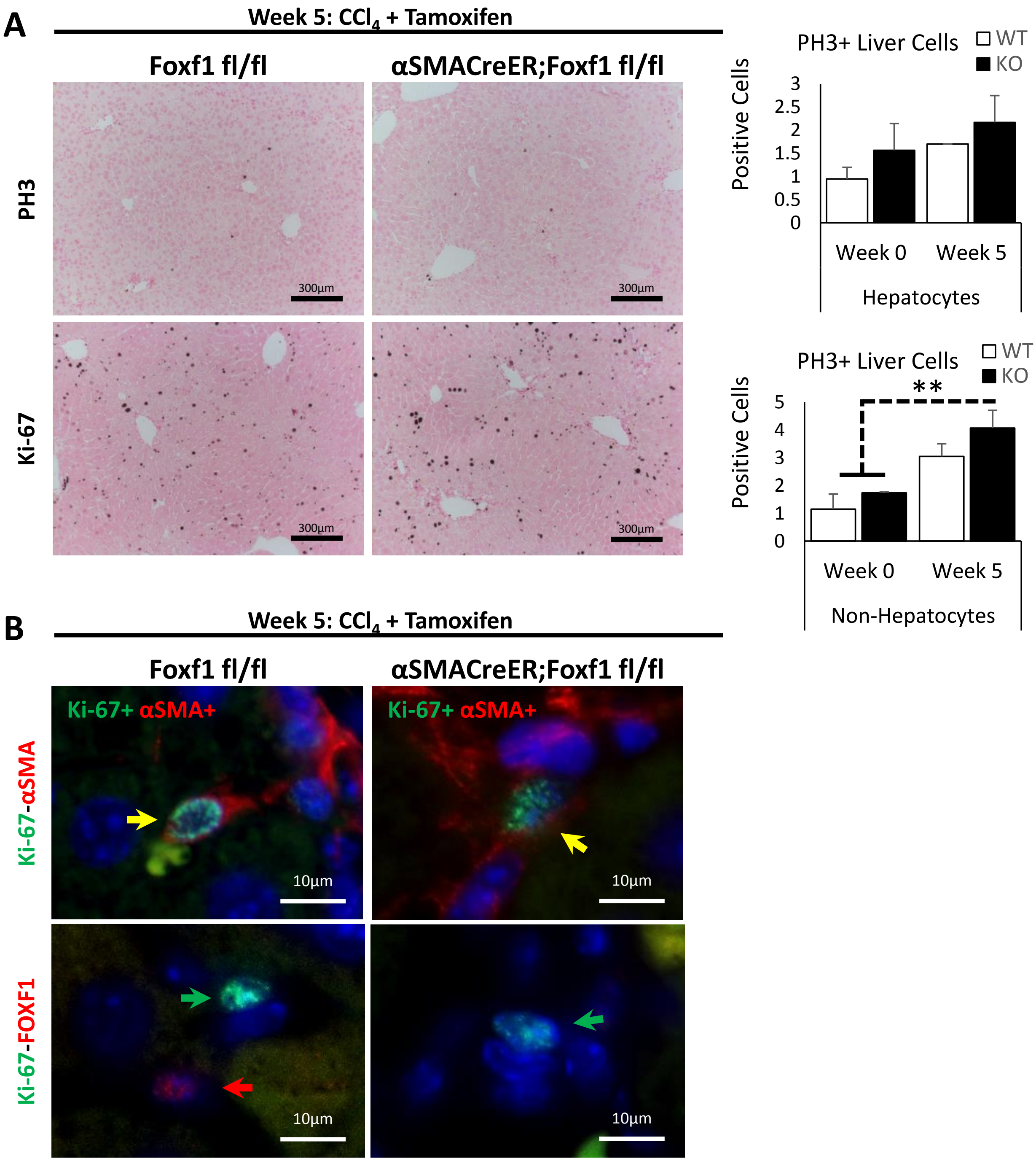

Figure S9. Deletion of Foxf1 *does not affect proliferation of* αSMA^{+} *cells in* CCl_4*-treated livers.* (A) Immunostaining for PH3 and Ki-67 and PH3 cell counts shows no difference between *Foxf1*$^{fl/fl}$ and *αSMACreER;Foxf1*$^{-/-}$ livers. The number of PH3^{+} hepatocytes and non-hepatocytes in *Foxf1*$^{fl/fl}$ livers was similar to those in *αSMACreER;Foxf1*$^{-/-}$ livers. Numbers of PH3^{+} cells were counted in 10 random 200x microscope fields using n=3 mice per group. (B) Co-localization of Ki-67 with αSMA shows the presence of Ki-67^{+} MFs in livers of CCl_4-treateed mice. Co-localization of Ki-67 with FOXF1 shows that proliferating FOXF1^{+} cells were uncommon in CCl_4-treated livers.

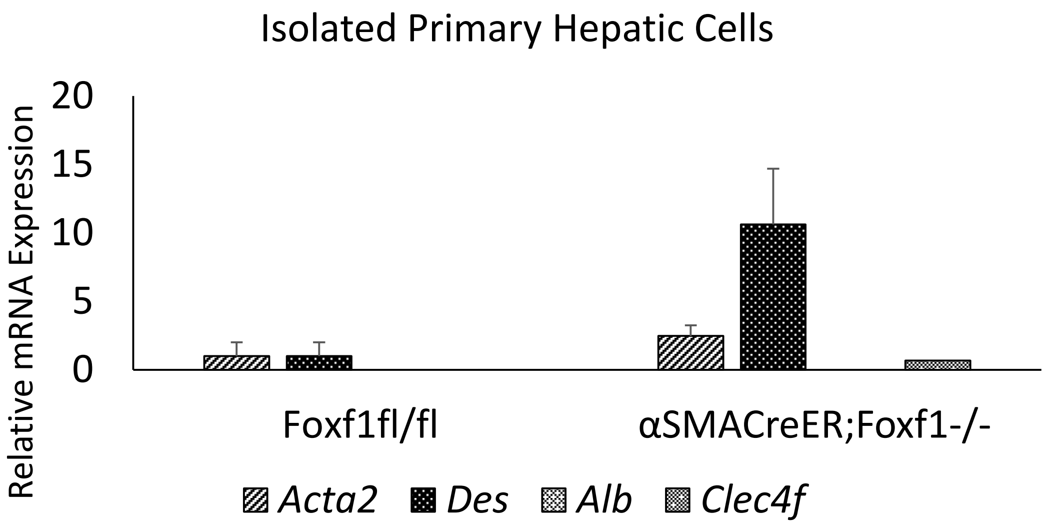

Figure S10. Purified stromal cells express Acta2 *and* Des. qRT-PCR analysis shows the presence of *Acta2* and *Des* mRNAs. Hepatocyte marker *Alb* and Kupffer cell marker *Clec4f* were not detected in purified stromal cells (one *αSMACreER;Foxf1*$^{-/-}$ sample expressed *Clec4f*). mRNAs were normalized to *Actb*.

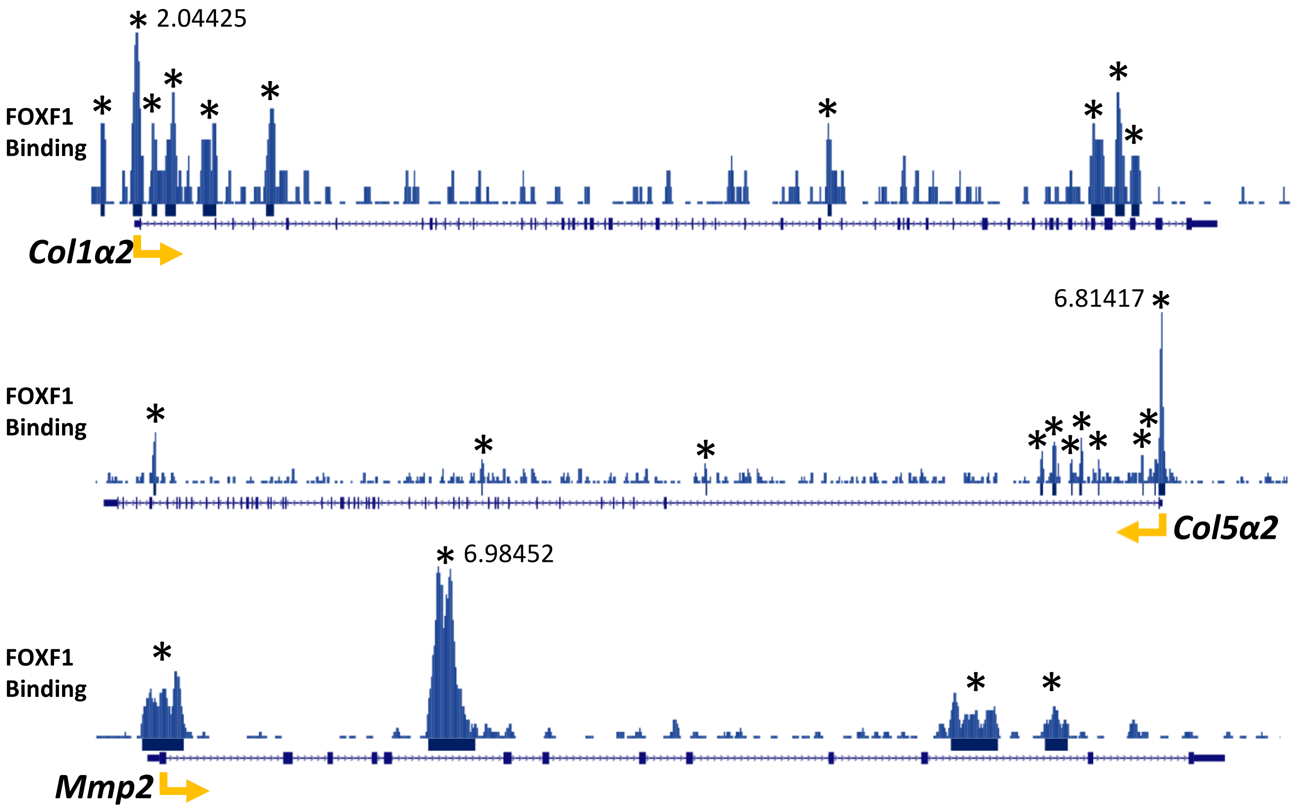

Figure S11. ChIP-seq shows FOXF1 binding sites in DNA regulatory regions of Col1α2, Col5α2, *and* Mmp2. Schematics of FOXF1 binding across entire genes: *Col1α2*, *Col5α2*, and *Mmp2*. ChIP-seq for FOXF1 shows significant binding in multiple DNA regions (indicated with *) of *Col1α2* (a peak binding height of 2.04425), *Col5α2* (a peak binding height of 6.81417), and *Mmp2* (a peak binding height of 6.98452).

Biology Open • Supplementary information

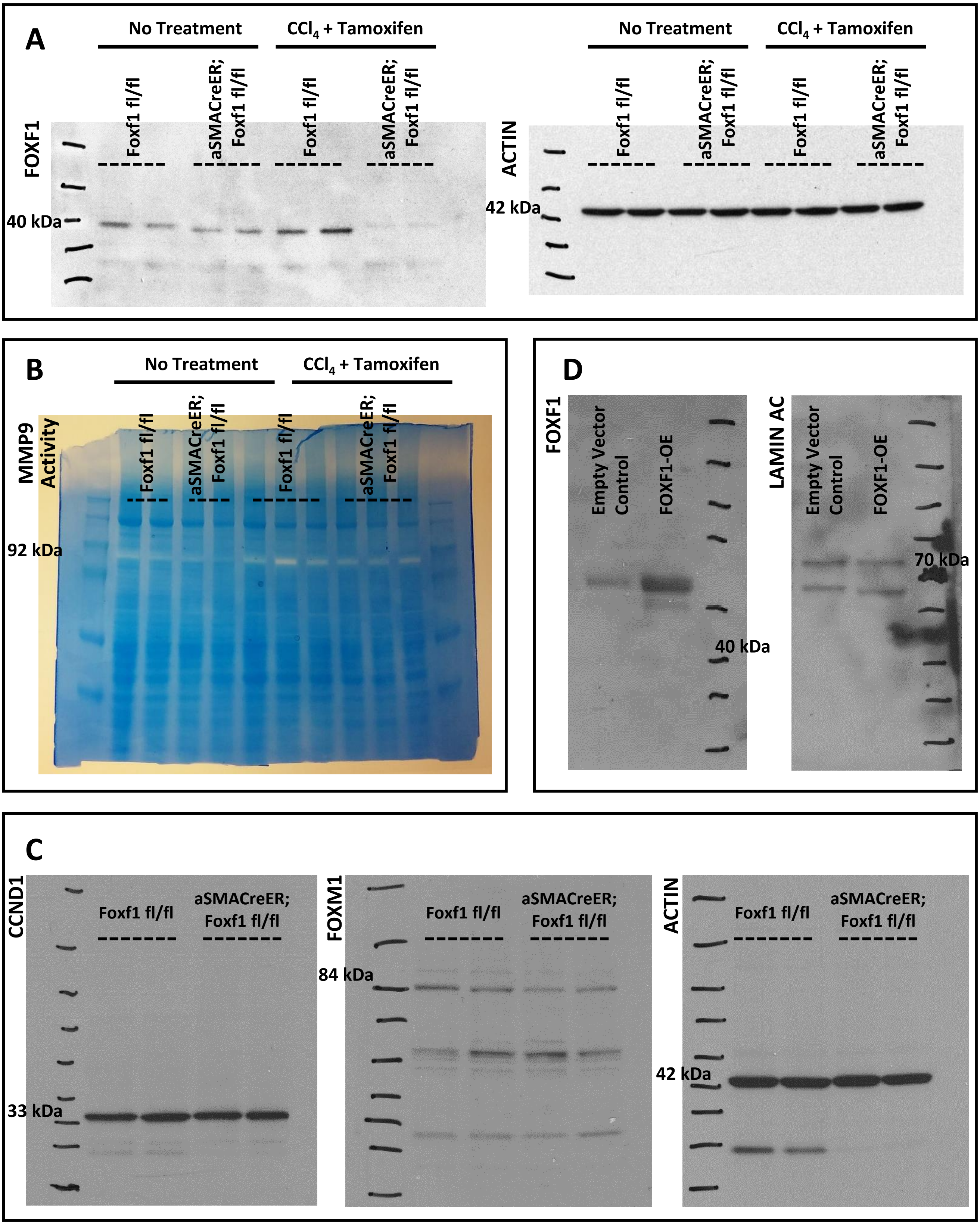
A
No Treatment
CCl4 + Tamoxifen
Foxf1 fl/fl
aSMACreER; Foxf1 fl/fl
Foxf1 fl/fl
aSMACreER; Foxf1 fl/fl
FOXF1
40 kDa
No Treatment
CCl4 + Tamoxifen
Foxf1 fl/fl
aSMACreER; Foxf1 fl/fl
Foxf1 fl/fl
aSMACreER; Foxf1 fl/fl
ACTIN
42 kDa
B
No Treatment
CCl4 + Tamoxifen
MMP9 Activity
Foxf1 fl/fl
aSMACreER; Foxf1 fl/fl
Foxf1 fl/fl
aSMACreER; Foxf1 fl/fl
92 kDa
D
FOXF1
Empty Vector Control
FOXF1-OE
40 kDa
LAMIN AC
Empty Vector Control
FOXF1-OE
70 kDa
C
CCND1
Foxf1 fl/fl
aSMACreER; Foxf1 fl/fl
33 kDa
FOXM1
Foxf1 fl/fl
aSMACreER; Foxf1 fl/fl
84 kDa
ACTIN
Foxf1 fl/fl
aSMACreER; Foxf1 fl/fl
42 kDa

Figure S12. Full images for Western blot and zymography. (A) Full Western blots for FOXF1 (40 kDa) and corresponding ACTIN (42 kDa). Samples derive from the same experiment and blots were processed in parallel. Cropped blots are shown in Fig. 2C. (B) Full zymography gel used for analysis of MMP9 activity (92 kDa). Cropped gel shown in Fig. 3G. (C) Full Western blots for CCND1 (33 kDa), FOXM1 (84 kDa), and corresponding ACTIN (42 kDa). Samples derive from the same experiment and blots were processed in parallel. Cropped blots shown in Fig. 4G. (D) Full Western blots for FOXF1 and corresponding LAMIN AC (70 kDa). The FOXF1 bands are higher than the predicted 40 kDa due to the tags on the vector. Cropped blots shown in Fig. 6E.
